# Architecture and development of olivocerebellar circuit topography

**DOI:** 10.3389/fncir.2012.00115

**Published:** 2013-01-02

**Authors:** Stacey L. Reeber, Joshua J. White, Nicholas A. George-Jones, Roy V. Sillitoe

**Affiliations:** ^1^Department of Pathology and Immunology, Baylor College of Medicine, Jan and Dan Duncan Neurological Research Institute of Texas Children's HospitalHouston, TX, USA; ^2^Department of Neuroscience, Baylor College of Medicine, Jan and Dan Duncan Neurological Research Institute of Texas Children's HospitalHouston, TX, USA

**Keywords:** inferior olive, circuitry, topography, climbing fibers, cerebellum, zones

## Abstract

The cerebellum has a simple tri-laminar structure that is comprised of relatively few cell types. Yet, its internal micro-circuitry is anatomically, biochemically, and functionally complex. The most striking feature of cerebellar circuit complexity is its compartmentalized topography. Each cell type within the cerebellar cortex is organized into an exquisite map; molecular expression patterns, dendrite projections, and axon terminal fields divide the medial-lateral axis of the cerebellum into topographic sagittal zones. Here, we discuss the mechanisms that establish zones and highlight how gene expression and neural activity contribute to cerebellar pattern formation. We focus on the olivocerebellar system because its developmental mechanisms are becoming clear, its topographic termination patterns are very precise, and its contribution to zonal function is debated. This review deconstructs the architecture and development of the olivocerebellar pathway to provide an update on how brain circuit maps form and function.

## Introduction

**Figure 1:**
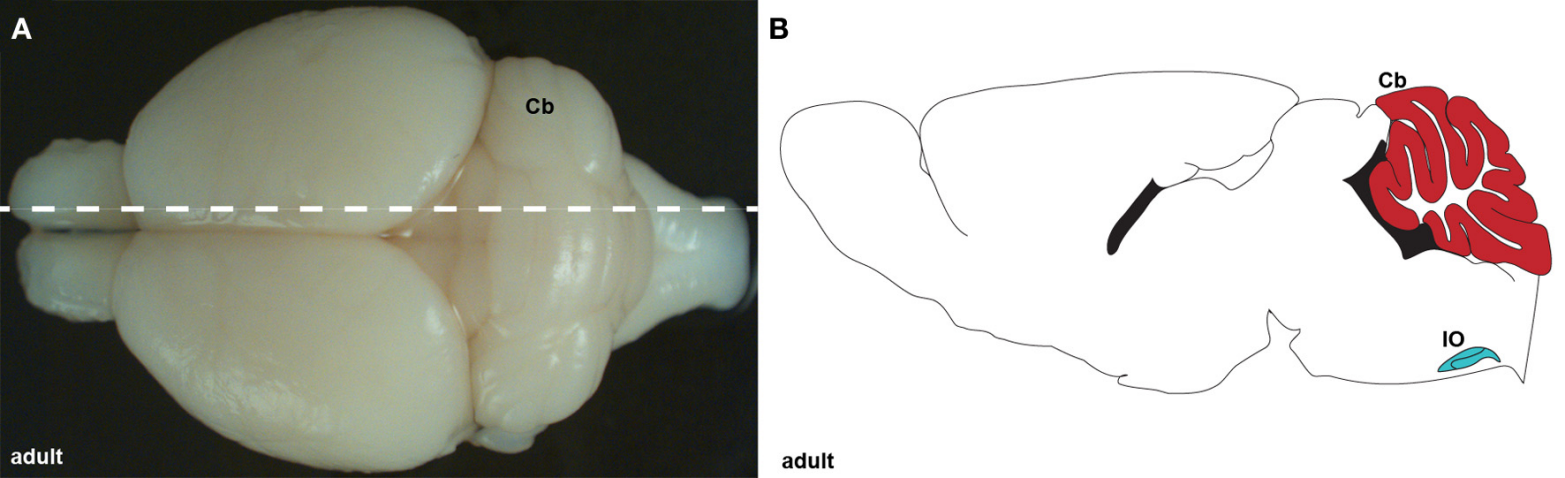
**(A)** Wholemount image of an adult brain showing the cerebellum (Cb) from a dorsal view. The dotted line indicates the level of the tissue section schematic in **(B)**. **(B)** Schematic of sagittal section cut through an adult cerebellum showing the cerebellum (red) and inferior olive (IO; blue).

**Figure 2:**
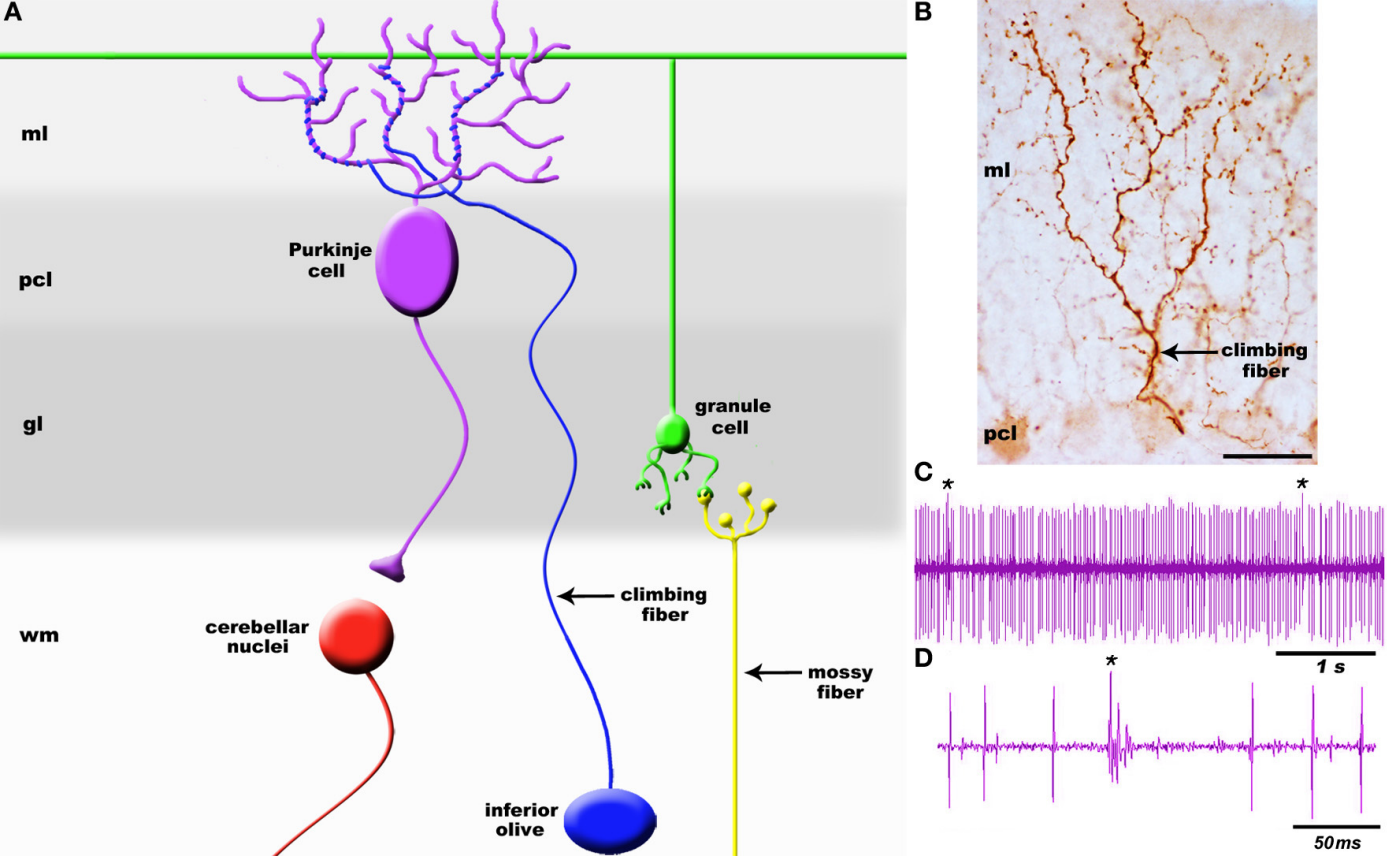
**(A)** Schematic of a simplified cerebellar microcircuit illustrating the two major sensory afferent pathways that project to the cerebellum: climbing fibers and mossy fibers. Climbing fibers (blue projection) terminate directly onto Purkinje cells whereas mossy fibers (yellow projection) terminate on granule cell dendrites (green). Granule cell axons called parallel fibers contact Purkinje cells (purple). Purkinje cells are the sole output of the cerebellar cortex and transmit signals to the cerebellar nuclei (red). **(B)** High power image of a climbing fiber expressing cocaine-and amphetamine-regulated transcript (CART) peptide [arrow; staining was performed according to Reeber and Sillitoe (2011)]. The target Purkinje cell is weakly immunoreactive for CART. **(C)** Example Purkinje cell spike train recorded *in vivo*. Recordings were performed in Ketamine/Xylazine anesthetized mice using 2–5 M Ohm Tungsten electrodes (Thomas Recording, Germany). Signals were band-pass filtered at 300–5000 Hz, amplified with an ELC-03XS amplifier (NPI, Germany), and recorded with Spike2 (CED, England). **(D)** Higher power view of the recording trace illustrating the clear distinction between a climbing fiber complex spike (cs) and simple spike (ss) responses in Purkinje cells. Asterisk in panel **(C)** indicates a complex spike. The layers of the cerebellum are indicated as molecular layer (ml), Purkinje cell layer (pcl), granular layer (gl), and white matter (wm). The cerebellar nuclei are located in the white matter. Scale bar in **(B)** = 25 μm.

It is well established that brain circuits are organized into spatial maps that control behavior (Hubel and Wiesel, 1979; Johnston, 1989; Friedman and O'Leary, 1996; Logan et al., 1996; Bozza et al., 2002; Huffman and Cramer, 2007; Leergaard and Bjaalie, 2007; Li and Crair, 2011; Suzuki et al., 2012). Yet, we have a limited understanding of how precise functional connections form during map development. Neural circuit connectivity is intensely studied in the cerebellum because its cellular networks are well understood and its developmental mechanisms are experimentally tractable. Cerebellar circuits have an established role in motor control and they are now also implicated in higher order functions such as cognition and emotion (Sacchetti et al., 2009; Strata et al., 2011). Two main types of afferents transmit information to the cerebellum: climbing fibers and mossy fibers. Climbing fibers arise only from neurons of the inferior olivary nucleus in the brainstem (Figure 1) and monoinnervate adult Purkinje cells (Figure 2A) whereas mossy fibers originate from numerous brain and spinal cord nuclei to innervate granule cells. Each climbing fiber elicits powerful Purkinje cell responses that sculpt cerebellar function (Figures 2C,D). Here, we discuss the development, organization, and function of the olivocerebellar projection and highlight the mechanisms that make this pathway an attractive model for understanding topographic brain circuitry.

## Cerebellar sagittal zones

**Figure 3:**
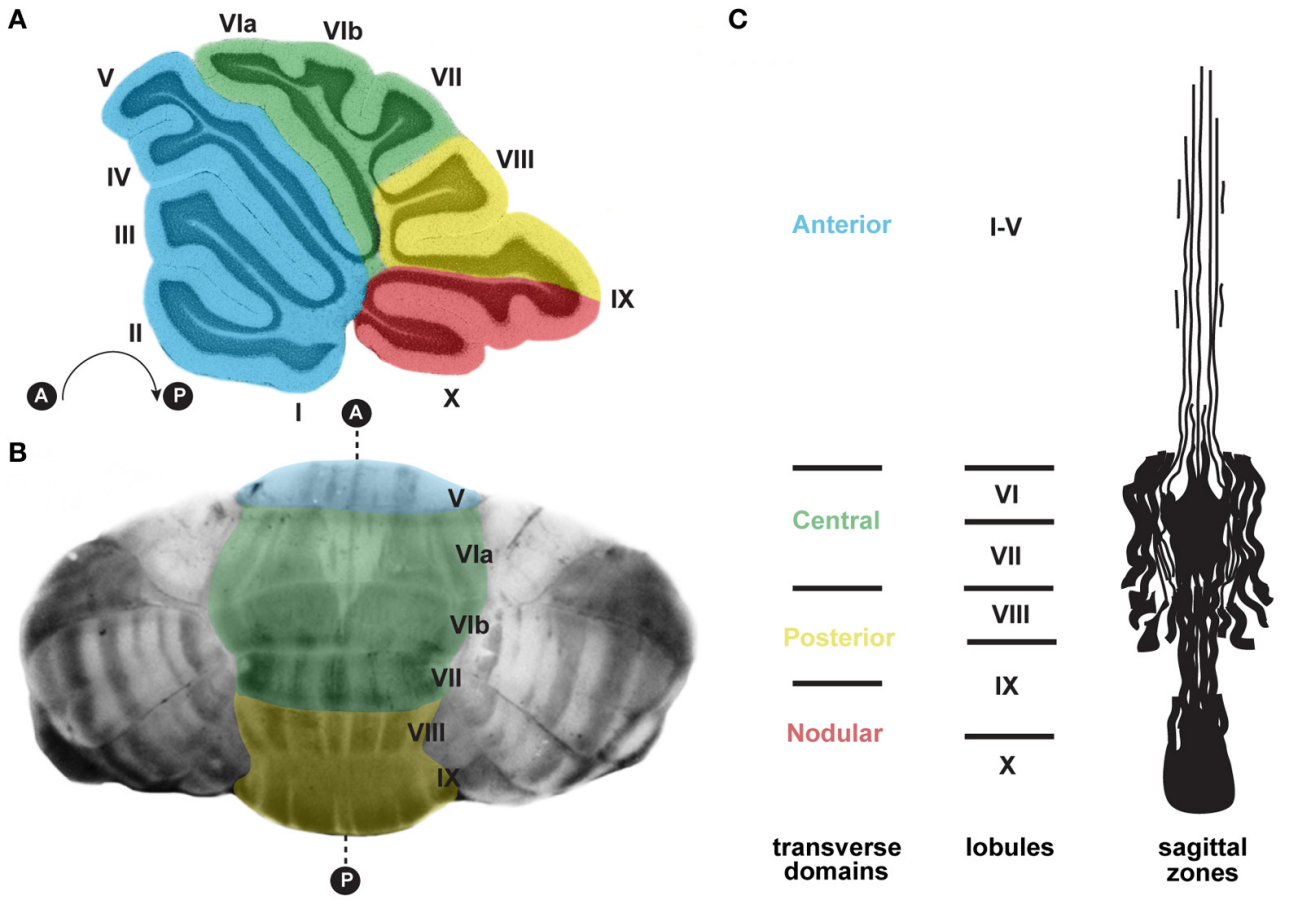
**(A)** Schematic of a sagittal section cut through the cerebellar vermis revealing the stereotypical foliation pattern, which consists of 10 lobules [adapted with permission from White and Sillitoe (2013)]. The cerebellum can be further divided along the anterior–posterior axis into four transverse domains: anterior (blue; lobules I–V), central (green; lobules VI and VII), posterior (yellow; lobules VIII and anterior IX), and nodular (red; lobules posterior IX and X) (Ozol et al., 1999). **(B)** In the adult cerebellum, zebrin II/aldolase C expression, which is revealed using wholemount staining (Sillitoe and Hawkes, 2002; White et al., 2012), delineates zones of Purkinje cells. The transverse zones are color coded according to panel **(A)**. **(C)** A schematic representation of an unfolded vermis illustrating the full pattern of zebrin II zones (adapted with permission from Sillitoe and Joyner, 2007). Lobule numbers are indicated by Roman numerals. Anterior and posterior axes are denoted by A and P.

The adult cerebellum is anatomically divided into distinct folds called lobules (Figure 3A; Larsell, 1952). Mammals and birds have 10 lobules that are separated from one another by a series of fissures. Because each fissure extends to a specific depth in the cerebellum, each lobule develops with a unique shape (Figure 3A). The invariance of lobule structure and their conservation across species support the idea that lobule/fissure formation is spatially and temporally controlled by complex morphogenetic programs (Sudarov and Joyner, 2007).

**Figure 4:**
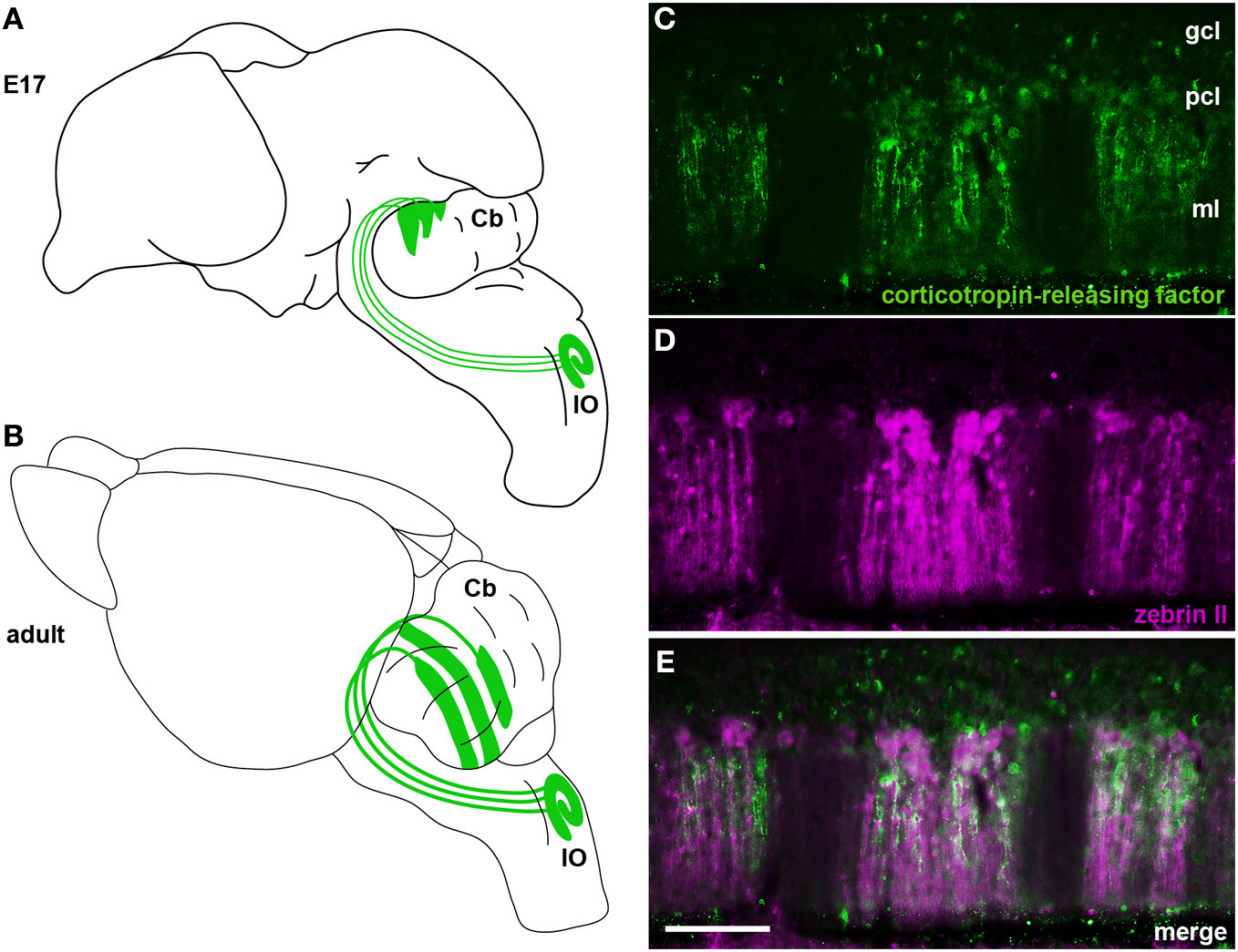
**(A)** Schematic illustrating climbing fibers projecting from the inferior olivary nucleus (IO) to the cerebellum (Cb) and their organization into a crude zonal pattern at E17. **(B)** A schematic of the adult brain showing climbing fibers projecting from the inferior olive (IO) to well-defined Purkinje cell zones in the cerebellum. **(C–E)** In the adult cerebellum corticotropin-releasing factor (CRF) is expressed in subsets of climbing fibers that align with zebrin II Purkinje cell zones. Panels **(C)** and **(D)** show individual channels of CRF and zebrin II expression and panel **(E)** is a merged image showing the corresponding relationship between the two patterns. The staining pattern of CRF and its relationship to zebrin II zones was previously described (Sawada et al., 2008). CRF and zebrin II staining was carried out exactly as previously described (Sawada et al., 2008). Scale bar in **(E)** = 100 μm (applies to **C–D**).

Strikingly, each lobule in the cerebellum is further compartmentalized along the medial-lateral axis into sagittal zones (Figure 3). Each set of zones is clearly delineated by the patterned expression of genes and proteins (Apps and Hawkes, 2009). The most comprehensively studied zonal marker is zebrin II (Brochu et al., 1990; Figures 3B,C, 4D), an antigen on the aldolase C protein (Ahn et al., 1994; Hawkes and Herrup, 1995). Zebrin II is expressed by alternating subsets of Purkinje cells (zebrin II+ adjacent to zebrin II−), thus forming complementary rows of biochemically distinct Purkinje cells (Figures 3B,C, 4D). The zonal organization of zebrin II is symmetrical about the cerebellar midline, highly reproducible between individuals, and conserved across species (Brochu et al., 1990; Sillitoe et al., 2005; Apps and Hawkes, 2009). The pattern of zebrin II has an intricate relationship to the expression of several other Purkinje cell proteins. For example, phospholipase Cβ3 (PLCβ3), sphingosine kinase 1a (SPHK1a), and excitatory amino-acid transporter 4 (EAAT4; Hawkes et al., 1985; Hawkes and Leclerc, 1987; Dehnes et al., 1998; Terada et al., 2004; Sarna et al., 2006) are all co-expressed with zebrin II. In contrast, phospholipase C β4 (PLCβ4; Armstrong and Hawkes, 2000; Sarna et al., 2006) is expressed selectively in zebrin II− zones. In addition to the complementary and corresponding relationships between zones, proteins such as neurofilament heavy chain (NFH) divide individual zebrin II zones into smaller sagittal units (Demilly et al., 2011). Cumulatively, molecularly defined zonal compartments divide the cerebellar cortex into hundreds of reproducible units with each one containing up to several hundred Purkinje cells (Apps and Hawkes, 2009).

Purkinje cell zones may be used to divide the cerebellum into four transverse domains in the anterior–posterior axis (Ozol et al., 1999). For example, in the vermis zebrin II expression reveals a specific pattern in lobules I–V and VIII/IX (Figures 3B,C, 4D). In contrast, expression of the small 25 kDa heat shock protein HSP25 delineates distinct zonal patterns in lobules VI/VII and IX/X, which express zebrin II in all Purkinje cells (Armstrong et al., 2000). Afferent termination patterns mirror the topography of Purkinje cell zones (Figures 4B,E). As a result, each transverse domain is innervated by a specific combination of functionally distinct afferent fibers. For instance, spinocerebellar mossy fibers project to lobules I–V and VIII/IX (Arsenio Nunes and Sotelo, 1985; Brochu et al., 1990; Sillitoe et al., 2010), whereas the vestibulocerellar mossy fibers project mainly to lobules IX and X (Jaarsma et al., 1997; Maklad and Fritzsch, 2003). In mouse, climbing fibers that express cocaine- and amphetamine-related transcript peptide (CART) terminate selectively in lobules VI/VII and IX/X (Reeber and Sillitoe, 2011), and corticotrophin releasing factor (CRF) expressing climbing fibers are expressed in a striking array of zones in lobules I–V and VIII/IX (Figures 4C,E) (Sawada et al., 2008).

The efferent side of the cortical circuit also respects the zonal topography. Sugihara and collaborators have mapped the trajectories of Purkinje cell axons from specific cerebellar cortical compartments onto the three sets of cerebellar nuclei. They revealed a close correspondence between adolase C expressing Purkinje cell terminals with subdivisions of cerebellar nuclei (Sugihara and Shinoda, 2007). Together, Purkinje cell zones, afferent topography, and Purkinje cell efferent projections to the cerebellar nuclei define the cerebellar module, the functional unit of the cerebellum (Apps and Hawkes, 2009; Ruigrok, 2011).

## Anatomical and functional organization of olivocerebellar zones

Fine topological mapping using anterograde tracers injected into specific sub-nuclei of the inferior olive and the tracing of climbing fiber collateral projections labeled from injections into the cerebellar cortex of birds, rodents, and primates have shown that there is a strict and precise association between climbing fiber topography and zebrin II Purkinje cell zones (Voogd et al., 2003; Sugihara and Shinoda, 2004; Voogd and Ruigrok, 2004; Sugihara and Quy, 2007; Pakan and Wylie, 2008; Sugihara et al., 2009; Fujita et al., 2010). In addition, several studies have used climbing fiber markers to link the architecture of chemically distinct subsets of climbing fiber afferents to the adult pattern of Purkinje cell zones (Table 1). For example, CRF, an amino acid peptide, is expressed in a subset of climbing fibers that corresponds to specific Purkinje cell zones (Sawada et al., 2001, 2008) (Figures 4C,E). In addition, we recently showed that the expression of the CART 55–102 peptide (Figure 2B) is intricately patterned into a complex topographic map that respects HSP25 (mouse) and zebrin II (rat) Purkinje cell zone boundaries (Reeber and Sillitoe, 2011). The class III intermediate filament protein peripherin is also expressed in a subset of climbing fibers that are organized into parasagittal compartments, although it is not clear how peripherin labeled climbing fibers relate to Purkinje cell zones (Errante et al., 1998). The precise topography of the olivocerebellar pathway raises the tantalizing possibility that zonal circuits may be functionally relevant. In this regard, two pressing questions have yet to be fully answered: (1) what is the functional significance of zones? and (2) what role do topographic circuits play during behavior?

**Table 1:** **Molecular and genetic markers for studying olivocerebellar topography**.

**Transient expression in subsets of climbing fibers**	
CGRP (zones in rat E16-P20)	Chedotal and Sotelo, 1992; Morara et al., 1992
Parvalbumin (zones in rat ~P0–P10)	Wassef et al., 1992; Chedotal and Sotelo, 1993
**Topographic climbing fiber projections**	
Calretinin (zones in cat)	Yan and Garey, 1996
CART (zones in mouse and rat)	Reeber and Sillitoe, 2011
CRF (zones in mouse and opossum)	Cummings et al., 1989; Sawada et al., 2008
DNPI/VGLUT2 (zones in mouse)	Paukert et al., 2010
NPY (zones in rat)	Ueyama et al., 1994
Peripherin (zones in rat)	Errante et al., 1998
**Compartmentalization of the inferior olive**	
BEN	Chedotal et al., 1996
Brn3a	Xiang et al., 1996
Brn3b	Xiang et al., 1996
CART	Reeber and Sillitoe, 2011
*Cdh6*	Suzuki et al., 1997
*Cdh8*	Suzuki et al., 1997; Redies et al., 2011
*Cdh11*	Suzuki et al., 1997
CRF	Yamano and Tohyama, 1994
*Cx36*	Belluardo et al., 2000; Weickert et al., 2005
*Cx45*	Van Der Giessen et al., 2006
*Cx47*	Weickert et al., 2005
C*x57*	Zappala et al., 2010
*DCC*	Bloch-Gallego et al., 1999
*EphA3*	Nishida et al., 2002
EPHA4	Hashimoto et al., 2012
*EphA5*	Nishida et al., 2002
*EphA6*	Nishida et al., 2002
EPHA7	Hashimoto et al., 2012
ER81	Zhu and Guthrie, 2002; Hashimoto et al., 2012
FOXP2	Hashimoto et al., 2012
NPY	Ueyama et al., 1994; Morara et al., 1997
Nr-CAM	Backer et al., 2002
*Pannexin1*	Weickert et al., 2005
*Pdh7*	Redies et al., 2011
*Pdh10*	Redies et al., 2011
*Unc-5H2*	Bloch-Gallego et al., 1999
*Unc-5H3*	Bloch-Gallego et al., 1999
DNPI/VGLUT2	Hisano et al., 2002
**Genetic markers for the inferior olive and/or climbing fibers**	
*CART-Cre*	Madisen et al., 2010
*CRF-Cre*	Martin et al., 2010
*Cx36-LacZ*	Degen et al., 2004
*Cx45-lacZ*	Van Der Giessen et al., 2006
*Npy-GFP*	Nishiyama et al., 2007
*Parvalbumin-Cre*	Tanahira et al., 2009
*Parvalbumin-CreER*	Taniguchi et al., 2011

*Note that the markers in each subsection are organized in alphabetical order and molecules of the same family are grouped together. The names of proteins are upper case and not italicized. mRNAs and transgenic mouse lines are italicized*.

Previous electrophysiological mapping studies suggested that parasagittal zones could be related to cerebellar function (Armstrong et al., 1974; Ekerot and Larson, 1980; Llinas and Sasaki, 1989; Chockkan and Hawkes, 1994; Sugihara et al., 1995; Chen et al., 1996; Hallem et al., 1999). However, it was only recently that modern optical imaging and electrophysiological approaches were exploited to uncover potential links between functional cerebellar circuits and zonal architecture (Ebner et al., 2012; Graham and Wylie, 2012). In their seminal paper, Wadiche and Jahr (2005) used molecular physiology approaches to demonstrate that synaptic plasticity may vary between zones. Accordingly, the level of glutamate that is released at climbing fiber terminals is zone dependent (Paukert et al., 2010) and climbing fiber inputs initiate synchronous firing in zones of Purkinje cells (Sasaki et al., 1989; Lang et al., 1999; Blenkinsop and Lang, 2006; Wise et al., 2010). These studies support the notion that there are fundamental differences in the physiology of Purkinje cell zones and suggest the possibility that climbing fibers contribute to the functional specificity of the zones.

The behavioral significance of zones remains elusive. However, surgically induced lesions and localized delivery of pharmacological agents into the inferior olive have provided some evidence that cerebellar zones may facilitate behavior (Watanabe et al., 1997; Seoane et al., 2005; Pijpers et al., 2008; Horn et al., 2010; Cerminara and Apps, 2011). For example, Llinas and collaborators found that when the neurotoxin 3-acetylpyridine (3AP) is injected intraperitoneally, the inferior olive is rapidly destroyed and severe ataxia emerges (Llinas et al., 1975). Similarly, inject-ing another neurotoxin called trans-crotononitrile (TCN) into rats inactivates the olive and induces profound motor deficits (Seoane et al., 2005; Cerminara and Apps, 2011). Ruigrok and colleagues used yet a different approach to inactivate the olive (Pijpers et al., 2008). They injected cholera toxin b conjugated to saporin into individual cerebellar cortical zones, which retrogradely transported the neurotoxin into the olive and induced dysfunction of specific modules. By targeting distinct modules they were able to demonstrated specific defects in the step phase-dependent modulation of cutaneously induced reflexes during locomotion (Pijpers et al., 2008; Cerminara and Apps, 2011). Moreover, inactivating specific olivary subdivisions in cats with the glutamate receptor blocker, CNQX, produced a series of unique motor deficits that were dependent on the particular sub-nucleus that was lesioned (Cerminara, 2010; Horn et al., 2010). What is far from clear is whether each zone encodes specific behaviors (or distinct aspects of a behavior), or whether multiple zones interact during motor control. Perhaps one way to unravel what zones do is to uncover how they form. Indeed, developmental studies have raised two critical questions that are ultimately relevant to cerebellar behavior: (1) what are the cellular and molecular mechanisms that control Purkinje cell zone development? and (2) how do climbing fiber projections invade, recognize, and connect to their targets?

## Genetic lineage, migration, and axonogenesis of inferior olive cells

Several landmark studies have used the regulatory sequences of developmentally expressed genes to design genetic tools for tracking the fate of cerebellar and inferior olive cells from embryogenesis to adulthood (Rodriguez and Dymecki, 2000; Hoshino et al., 2005; Machold and Fishell, 2005; Pascual et al., 2007). Genetic fate-mapping studies using *Atonal homolog 1* (*Atoh1*, formerly known as *Math1)* and *Wnt1* regulatory elements revealed that inferior olive neurons emerge from a distinct progenitor pool in the lower rhombic lip of the hindbrain (Rodriguez and Dymecki, 2000; Landsberg et al., 2005; Wang et al., 2005; Nichols and Bruce, 2006). In accordance with these findings, genetic fate-mapping using a *pancreas specific transcription factor 1a*-*Cre* (*Ptf1a^Cre/+^*) allele to drive *lacZ* reporter gene expression in *R26R* [*Gt*(*ROSA*)*26Sor^tm1sor^*; Soriano, 1999] mice revealed that inferior olivary neurons are derived from a distinct *Ptf1a* domain (Hoshino et al., 2005; Yamada et al., 2007). Hoshino and colleagues determined that *Ptf1a* is required for the proper development of inferior olive neurons, because the inferior olivary complex is severely altered in *Ptf1a* null mutants (Yamada et al., 2007). Without *Ptf1a*, some inferior olive neurons do not differentiate while others migrate inappropriately. Moreover, a large number of apoptotic cells were observed in the *Ptf1a* mutants, and the fate of *Ptf1a*-dependent lineages adopted mossy fiber neuron characteristics (Yamada et al., 2007). Although *Ptf1a* appears to control the development of most, if not all, olivary neurons, it is not clear what upstream or downstream molecular pathways might be responsible for generating the sub-nuclei. Studies by Bloch-Gallego and colleagues provide some insight into this question. The authors determined that the absence of Rho-guanine exchange factor Trio impairs the organization of the inferior olivary nucleus into distinct lamellae (Backer et al., 2007). Additionally, in a recent elegant study, quail-chick chimaeras were used to provide evidence that each inferior olive sub-nucleus originates from specific rhombomeres, developmental hindbrain units that are each restricted in their lineages (Hidalgo-Sanchez et al., 2012). It is intriguing that climbing fiber zones, which arise from distinct olivary sub-nuclei, may be specified early by rhombomere specific cues.

Inferior olive neurons are born dorsally in the lower rhombic lip and migrate circumferentially around the edges of the brainstem to their final location near the ventral midline (Altman and Bayer, 1987; Sotelo, 2004; Sotelo and Chedotal, 2005) (Table 2). Tritiated thymidine labeling (Altman and Bayer, 1987) and HRP axonal tracing *in vitro* (Bourrat and Sotelo, 1988, 1990b) revealed that inferior olivary neurons migrate along the lateral edges of the brainstem in a unique “submarginal stream” (Altman and Bayer, 1987; Bourrat and Sotelo, 1988, 1990b; Sotelo and Chedotal, 2005). Interestingly, the somata of olivary neurons do not cross the floor plate, whereas their axons do cross and project exclusively to the contralateral cerebellum (Altman and Bayer, 1987; Altman, 1997). The restriction of olivary neurons to one side of the midline is controlled by both chemoattractive and chemorepellent molecules (e.g., netrin-1/DCC and Slit/Robo; Bloch-Gallego et al., 1999; Causeret et al., 2002; de Diego et al., 2002; Marillat et al., 2004). Marillat et al. (2004) showed that Rig-1/Robo3 plays an essential role in controlling the migration of precerebellar neurons and the projection of axons across the midline. In *Rig1/Robo3* deficient mice, inferior olive neurons incorrectly send axons to the ipsilateral cerebellum in addition to sending the normal contralateral projection (Marillat et al., 2004).

**Table 2:** **Timeline of olivocerebellar development**.

**Developmental stage**	**Developmental event**	**References**
~E12/13 rat (E10/11 mouse)	Inferior olive neurons are born	Pierce, 1973; Bourrat and Sotelo, 1990a, 1991; Sotelo, 2004
~E14/15 mouse	Climbing fibers arrive in cerebellum	Paradies and Eisenman, 1993
~E16–E18 rat (E14–16 mouse)	Inferior olive neurons settle in final position adjacent to the floor plate	Bourrat and Sotelo, 1990a; Sotelo, 2004
~E16 rat (E14 mouse)	Transient biochemical compartmentation of inferior olive and Purkinje cells (arising independently)	Wassef et al., 1992; Larouche et al., 2006
~E15/16 mouse	Climbing fibers organize into crude parasagittal clusters	Paradies and Eisenman, 1993
~E17 mouse	Climbing fiber topography corresponds clearly with nascent Purkinje cell zone	Paradies et al., 1996
~P0–P5 rat (P0–P3 mouse)	Olivocerebellar projections resolve into precise sagittal zones similar to the adult	Sotelo et al., 1984
~P0 rat (P0 mouse)	Creeper stage starts	Watanabe and Kano, 2011
~P0–P10 rat (P0–P8 mouse)	Critical period for olivocerebellar plasticity	Sherrard et al., 1986
~P3 mouse	Discrete climbing fiber mediated EPSCs recorded in Purkinje cells (all fibers induce similar amplitudes in perinatal Purkinje cells)	Hashimoto and Kano, 2003
~P5 rat (P3 mouse)	Pericellular nest stage starts	Watanabe and Kano, 2011
~P5 mouse	Development of climbing fiber terminal structure	Mason and Gregory, 1984
~P7 mouse	“Winner” climbing fiber is strengthened	Hashimoto and Kano, 2003
End of the first postnatal week	Climbing fiber complex spikes are first detected	Woodward et al., 1969
~P9 rat (P7 mouse)	Capuchon stage	Watanabe and Kano, 2011
~P12 rat (P10 mouse)	Dendritic stage commences	Watanabe and Kano, 2011
~P7–11 rat (P5–9 mouse)	Climbing fiber pruning and perisomatic synpase elimination: the early phase	Watanabe and Kano, 2011
~P12–17 rat (P10–15 mouse)	Climbing fiber pruning and perisomatic synpase elimination: the late phase	Watanabe and Kano, 2011

The first climbing fibers arrive in the developing cerebellum at ~embryonic day (E) 14/15 in the mouse (Paradies and Eisenman, 1993) (Table 2) and are already organized in a crude zonal map at ~E15/16 (Sotelo et al., 1984; Chedotal and Sotelo, 1992; Paradies and Eisenman, 1993; Paradies et al., 1996), which is approximately when Purkinje cells begin to express parasagittal markers (e.g., *engrailed1/2* and *L7/Pcp2*) (Hashimoto and Mikoshiba, 2003; Wilson et al., 2011). By ~E17 in mice, olivocerebellar topography strongly corresponds with the nascent architecture of Purkinje cell zones (Paradies et al., 1996; Figure 4A).

## Formation of olivocerebellar zones

The almost perfect overlap between climbing fiber terminal field topography and Purkinje cell zones suggests that the spatial and temporal targeting of cerebellar afferent pathways is closely coordinated with Purkinje cell development. Purkinje cells become postmitotic between ~E10 and ~E13 and form symmetrical zonal “clusters” by ~E14 (Hashimoto and Mikoshiba, 2003; Hoshino et al., 2005; Sillitoe et al., 2009; Namba et al., 2011; Sudarov et al., 2011). Climbing fiber neurons are also born at ~E10/11 (Sugihara and Shinoda, 2007). Interestingly, when they arrive in the developing cerebellum they immediately project into clusters of Purkinje cells (Paradies and Eisenman, 1993). The predictable termination of climbing fibers into Purkinje cell zones suggests that the Purkinje cells may play an active role in instructing the pattern of olivocerebellar targeting.

Sotelo and collaborators postulated that the cerebellum and the inferior olive might have matching gene expression domains that establish bidirectional signaling to generate the olivocerebellar map (Sotelo and Wassef, 1991; Sotelo and Chedotal, 1997, 2005). Support for this hypothesis was first provided by using a combination of markers that labeled zones of Purkinje cells (calbindin, GMP-cyclic dependent protein kinase, Purkinje cell-specific glycoprotein, and PEP-19) and also marked corresponding subsets of inferior olive cells along with their projections [calbindin, parvalbumin, and calcitonin gene-related peptide (CGRP); Table 1]. The precision and reproducibility of zonal boundaries defined by these markers suggested the possibility that inferior olivary neurons might target Purkinje cell zones by recognizing positional cues (Sotelo and Wassef, 1991; Sotelo and Chedotal, 1997, 2005).

Eph/ephrin genes play a major role in establishing brain topography (Flanagan and Vanderhaeghen, 1998; Cang et al., 2008a,b; Allen-Sharpley and Cramer, 2012). In the cerebellum, eph/ephrin are expressed in distinct parasagittal domains (Karam et al., 2000, 2002). Nishida and coworkers (2002) provided compelling evidence for the involvement of eph/ephrin signaling in controlling the molecular matching between climbing fibers and Purkinje cells during olivocerebellar circuit formation (Nishida et al., 2002). They showed that altering ephA receptor and ephrin-A ligand expression in chick hindbrain explant cultures disrupted the anterior–posterior targeting of olivocerebellar axons. However, this study did not address whether eph/ephrin signaling controls the development of olivocerebellar zones (Nishida et al., 2002; Hashimoto and Hibi, 2012). Regardless, because specific ephA/ephrin-A manipulations can disrupt the global targeting of olivocerebellar axons, there is a possibility that other eph/ephrins and/or additional molecules likely cooperate to establish precise Purkinje cell-afferent interactions during map formation. Besides the eph/ephrins, possible candidates are the type-II classic cadherin and δ-protocadherin cell–cell adhesion molecules, which are expressed in a striking array of Purkinje cell sagittal zones (Suzuki et al., 1997; Neudert et al., 2008; Redies et al., 2011) and in specific subdivisions of the inferior olive (Suzuki et al., 1997; Neudert et al., 2008; Redies et al., 2011). Despite these clues, we still do not have a clear picture of what genes control the topographic connectivity of olivocerebellar zones nor do we understand the detailed mechanisms that initiate and maintain the physical interaction between specific Purkinje cells and climbing fibers. However, recent work demonstrates that starting from birth, inferior olive neurons spontaneously organize into clusters that fire synchronous Ca^2+^ transients in *in vitro* brain slice preparations (Rekling et al., 2012). Curiously, during early postnatal development spontaneous waves travel along chains of axon collaterals that connect sagittal rows of Purkinje cells (Watt et al., 2009). Both phenomena were suggested as likely mechanisms contributing to the development of cerebellar compartments. However, whether the spontaneous waves of Purkinje cell activity are linked to the spontaneous activity of inferior olive neurons awaits further analysis. It will also be interesting to determine whether cerebellar spontaneous activity interacts with developmental gene function in a zone specific fashion.

## Postnatal remodeling of climbing fibers

Following the establishment of the crude zonal map, climbing fibers undergo extensive morphological changes and proceed through different stages of fiber remodeling to form functionally mature connections (Watanabe and Kano, 2011) (Table 2). The first phase of remodeling is the “creeper” stage (~P0 in rat) when climbing fibers are very thin and form transient synapses on immature Purkinje cell dendrites (Chedotal and Sotelo, 1993; Sugihara, 2005; Watanabe and Kano, 2011). Then, climbing fibers enter a “transitional” stage and exhibit characteristics that are intermediate between those of the creeper and nest stages (Sugihara, 2005). The “pericellular nest' stage (~P5) is defined by the dense terminal arbors (“nest”) that surround Purkinje cell somata (Cajal, 1911; O'Leary et al., 1971; Mason et al., 1990; Sugihara, 2005; Watanabe and Kano, 2011). During this stage, each Purkinje cell receives polyneuronal input from more than five different climbing fibers. Climbing fibers are progressively displaced onto the developing dendritic stems of maturing Purkinje cells (“capuchon stage”; starting at ~P9). As the dendritic arbors develop, the climbing fibers leave their perisomatic and capuchon positions to occupy peridendritic positions (after ~P12; referred to as dendritic stage; Chedotal and Sotelo, 1992; Watanabe and Kano, 2011). During this period, climbing fibers translocate up the Purkinje cell dendrite to find their ultimate location within the basal two thirds of the molecular layer (Crepel et al., 1976; Mariani and Changeux, 1981; Hashimoto and Kano, 2005; Kano and Hashimoto, 2009; Watanabe and Kano, 2011).

The monoinnervation of adult climbing fibers onto Purkinje cells is achieved through massive pruning of climbing fibers during postnatal development. Previous studies have revealed systematic changes occurring in the relative synaptic strength of multiple climbing fibers when they polyinnervate a single Purkinje cell during postnatal development. These studies revealed that climbing fiber mediated excitatory postsynaptic currents (EPSCs) recorded in Purkinje cells have similar amplitudes until ~P3. In the second postnatal week, multiple EPSCs differentiate into one large EPSC and a few small EPSCs (Hashimoto and Kano, 2003). These results suggest that climbing fiber synaptic strengths are similar to one another during early postnatal development, and a single climbing fiber, the “winner,” is selectively strengthened during the second postnatal week (~P7; Hashimoto and Kano, 2003; Bosman et al., 2008). Following these studies, Kano and colleagues used electrophysiological and morphological techniques to determine that competition between multiple climbing fibers occurs at the soma before climbing fibers form synapses with Purkinje cell dendrites (Hashimoto et al., 2009). Notably, the “winner” climbing fiber undergoes translocation to the dendrites and simultaneously maintains synapses on the soma, while the weaker climbing fibers remain around the soma forming “pericellular nests” with the “winner” synapses (Hashimoto et al., 2009). After the strengthening of a single “winner” climbing fiber, pruning and perisomatic synapse elimination occur in two distinct phases: the early phase (~P7–11), which is independent of parallel fiber synapses and the late phase (~P12–17), which depends on activity between parallel fibers and Purkinje cells (Watanabe and Kano, 2011).

In three different mutant mice, *weaver*, *staggerer*, and *reeler*, Purkinje cells develop in the absence of granule cells but are permanently innervated by multiple climbing fibers (Crepel and Mariani, 1976; Mariani et al., 1977; Crepel et al., 1980; Mariani and Changeux, 1980; Steinmayr et al., 1998). Similarly, studies using experimentally-induced “hypogranular” cerebella (Woodward et al., 1974; Crepel and Delhaye-Bouchaud, 1979; Bravin et al., 1995; Sugihara et al., 2000) revealed that the presence of intact granule cells, normal parallel fiber-Purkinje cell synapses, and activity all play a role in climbing fiber synapse elimination.

The process of fiber elimination is mediated by several molecules including metabotropic glutamate receptor mGluR1, PLCβ4, Ca(v)2.1 P/Q-type Ca^2+^ channel, glutamate receptor Glurδ2, precerebellin (or Cbln1), and the GABA synthesizing enzyme GAD67 (Kano et al., 1995, 1997, 1998; Kashiwabuchi et al., 1995; Offermanns et al., 1997; Sugihara et al., 1999; Ichikawa et al., 2002; Miyazaki et al., 2004, 2010; Hirai et al., 2005; Uemura et al., 2007; Hashimoto et al., 2011; Nakayama et al., 2012; Uesaka et al., 2012). Mutations that alter the function of these proteins cause severe defects in climbing fiber synapse development and elimination (Kano et al., 1995, 1997, 1998; Kashiwabuchi et al., 1995; Offermanns et al., 1997; Sugihara et al., 1999; Ichikawa et al., 2002; Miyazaki et al., 2004, 2010; Hirai et al., 2005; Uemura et al., 2007; Hashimoto et al., 2011; Nakayama et al., 2012; Uesaka et al., 2012). Interestingly, Kano and colleagues developed an organotypic co-culture preparation to recapitulate *in vivo* climbing fiber remodeling and with this system identified neuroligin-2 as a key player of climbing fiber elimination in Purkinje cells (Uesaka et al., 2012). Thus, synaptogenesis in the olivocerebellar projection starts relatively early during brain circuit formation, occurs over a protracted period of time, and requires both genetic control and neural activity (Chedotal and Sotelo, 1992; Sotelo, 2004). However, it is not clear whether developmental remodeling plays a role in generating climbing fiber compartments: although one can imagine that the precise zonal boundaries emerge as supernumerary axons are pruned away.

## Plasticity of olivocerebellar zone connectivity

In contrast to the adult central nervous system which has a limited capacity for axonal regeneration, the immature central nervous system is capable of some axonal regrowth (Nicholls and Saunders, 1996). However, regrowth during development frequently occurs through an alternative pathway that is distinct from the normal one. The olivocerebellar pathway is an excellent example of a system in which regrowth establishes a new pathway. Various groups have used the pedunculotomy approach to stimulate transcommissural olivocerebellar reinnervation to determine the temporal properties of afferent-target interactions during development (Angaut et al., 1985; Sherrard et al., 1986; Zagrebelsky et al., 1997; Sugihara et al., 2003; Dixon et al., 2005; Willson et al., 2007). Following unilateral early postnatal transection of an inferior cerebellar peduncle (which carries the climbing fibers), the contralateral inferior olive degenerates and new axons, arising from the remaining inferior olive, grow into the denervated hemicerebellum (Zagrebelsky et al., 1997). The innervation of these transcommissural axons precisely aligns with Purkinje cell expression zones and mirrors the distribution of the “unaltered” projections on the intact side (Zagrebelsky et al., 1997). Sugihara and colleagues (2003) have shown that the newly formed projections develop normal climbing fiber arborizations and form functional synapses onto Purkinje cells. Remarkably, olivocerebellar reinnervation can compensate for motor deficits (Dixon et al., 2005) and rescue the cerebellums influence over spatial learning (Willson et al., 2007). Similar to what might occur during normal development, reinnervation may be regulated by position-dependent cues that mediate the precise connectivity between climbing fibers and Purkinje cells (Dixon and Sherrard, 2006; Willson et al., 2008).

## Novel tools to study olivocerebellar development, connectivity, and function

Neuronal tracing using viruses and genetically encoded fluorescent reporters are now widely used for unraveling circuit connectivity (Wickersham et al., 2007; Marshel et al., 2010; Wall et al., 2010). Retrograde transneuronal infection of rabies virus reveals the organization of multi-synaptic neuronal networks (Coulon et al., 1989; Ugolini, 1995; Kelly and Strick, 2000; Graf et al., 2002). Genetically modified viruses have also allowed control over which cells are initially infected, extent of viral spread, and direction of the spread (Callaway, 2008). Recently, the use of a deletion-mutant rabies virus allowed the spread of the virus to be restricted to monosynaptic connections for selectively revealing first-order presynaptic neurons (Wickersham et al., 2007, 2010; Marshel et al., 2010; Rancz et al., 2011). Using the rabies virus tracing approach, communication networks between the cerebral cortex, basal ganglia, and cerebellum have been resolved (Kelly and Strick, 2003; Bostan et al., 2010; Coffman et al., 2011; Suzuki et al., 2012). More recently, Ruigrok and colleagues also used viral tracing to show that cerebrocerebellar connectivity respects cerebellar zonal organization (Suzuki et al., 2012). Combining viral tracing with transgenic targeting of recombinant viruses (Weible et al., 2010) will allow for unparalleled resolution of circuit topography in the olivocerebellar pathway.

In the past, lesioning, electrical stimulation, and chemical activation/deactivation have unveiled essential functions of the cerebellum and inferior olive (Llinas et al., 1975; McCormick and Thompson, 1984; Bradley et al., 1991; O'Hearn et al., 1993; O'Hearn and Molliver, 1993; Willson et al., 2007; Pijpers et al., 2008; Strick et al., 2009; Horn et al., 2010; Cerminara and Apps, 2011). However, these manipulations are limited by the lack of cell type specificity and/or the by the tissue damage that occurs. Optogenetics methods offer an ideal solution to these shortcomings as they provide an avenue for targeting induced neural activity to specific cells *in vivo*, without damaging the circuit (Deisseroth et al., 2006; Zhang et al., 2006; Hira et al., 2009; Tsubota et al., 2011). These light-activated ion channels, which include channelrhodopsin-2 (ChR2) and halorhodopsin (eNpHR), have fast temporal kinetics to efficiently activate or inhibit the firing of action potentials (Boyden et al., 2005; Zhang et al., 2006; Adamantidis et al., 2007; Arenkiel et al., 2007; Abbott et al., 2009). Importantly, by using cell type specific promoters one can drive the expression of these light-responsive proteins in selective neuronal populations (e.g., using the *L7/Pcp2* Purkinje cell specific promoter; Oberdick et al., 1990). Indeed, a recent study used *L7/Pcp2-Cre* mice to target ChR2 and eNpHR expression to examine the role of Purkinje cells in controlling cardiovascular function (Tsubota et al., 2011). It will now be interesting to develop optogenetic methods for manipulating neuronal activity within specific inferior olivary nuclei in order to determine the contribution of olivocerebellar zones to motor and nonmotor functions *in vivo*.

## Summary

It is well established that the cerebellum is divided into a complex map of functional zones. Much progress has been made in delineating the zonal topography between the inferior olivary nucleus, cerebellar cortex, and the cerebellar nuclei. However, there are several important questions that remain unanswered. For example: (1) Are the olivocerebellar cells that project to each cerebellar zone born at different times and/or are they derived from different genetic lineages? (2) What are the molecular mechanisms that guide olivocerebellar projections into zonal compartments? and (3) What behaviors are encoded into each zone? In future studies, it will be interesting to combine modern anatomical tracing techniques with high-resolution imaging, sophisticated genetic approaches and electrophysiology to answer such questions.

### Conflict of interest statement

The authors declare that the research was conducted in the absence of any commercial or financial relationships that could be construed as a potential conflict of interest.

## References

[B1] Abbott S. B. Stornetta R. L. Socolovsky C. S. West G. H. Guyenet P. G. (2009). Photostimulation of channelrhodopsin-2 expressing ventrolateral medullary neurons increases sympathetic nerve activity and blood pressure in rats. J. Physiol. 587, 5613–5631 10.1113/jphysiol.2009.177535 19822543 PMC2805374

[B2] Adamantidis A. R. Zhang F. Aravanis A. M. Deisseroth K. de Lecea L. (2007). Neural substrates of awakening probed with optogenetic control of hypocretin neurons. Nature 450, 420–424 10.1038/nature06310 17943086 PMC6744371

[B3] Ahn A. H. Dziennis S. Hawkes R. Herrup K. (1994). The cloning of zebrin II reveals its identity with aldolase C. Development 120, 2081–2090 7925012 10.1242/dev.120.8.2081

[B5] Allen-Sharpley M. R. Cramer K. S. (2012). Coordinated Eph-ephrin signaling guides migration and axon targeting in the avian auditory system. Neural Dev. 7:29 10.1186/1749-8104-7-29 22908944 PMC3515360

[B6] Altman J. Bayer S. A. (1987). Development of the precerebellar nuclei in the rat: IV. The anterior precerebellar extramural migratory stream and the nucleus reticularis tegmenti pontis and the basal pontine gray. J. Comp. Neurol. 257, 529–552 10.1002/cne.902570405 3693597

[B7] Altman J. B. S. (1997). Development of the Cerebellar System in Relation to its Evolution, Structure, and Functions. New York, NY: CRC

[B8] Angaut P. Alvarado-Mallart R. M. Sotelo C. (1985). Compensatory climbing fiber innervation after unilateral pedunculotomy in the newborn rat: origin and topographic organization. J. Comp. Neurol. 236, 161–178 10.1002/cne.902360203 2414330

[B9] Apps R. Hawkes R. (2009). Cerebellar cortical organization: a one-map hypothesis. Nat. Rev. Neurosci. 10, 670–681 10.1038/nrn2698 19693030

[B10] Arenkiel B. R. Peca J. Davison I. G. Feliciano C. Deisseroth K. Augustine G. J. (2007). *In vivo* light-induced activation of neural circuitry in transgenic mice expressing channelrhodopsin-2. Neuron 54, 205–218 10.1016/j.neuron.2007.03.005 17442243 PMC3634585

[B11] Armstrong C. L. Hawkes R. (2000). Pattern formation in the cerebellar cortex. Biochem. Cell Biol. 78, 551–562 11103945

[B12] Armstrong C. L. Krueger-Naug A. M. Currie R. W. Hawkes R. (2000). Constitutive expression of the 25-kDa heat shock protein Hsp25 reveals novel parasagittal bands of purkinje cells in the adult mouse cerebellar cortex. J. Comp. Neurol. 416, 383–397 10.1002/(SICI)1096-9861(20000117)416:3<383::AID-CNE9>3.0.CO;2-M 10602096

[B13] Armstrong D. M. Harvey R. J. Schild R. F. (1974). Topographical localization in the olivo-cerebellar projection: an electrophysiological study in the cat. J. Comp. Neurol. 154, 287–302 10.1002/cne.901540305 4826097

[B14] Arsenio Nunes M. L. Sotelo C. (1985). Development of the spinocerebellar system in the postnatal rat. J. Comp. Neurol. 237, 291–306 10.1002/cne.902370302 3840179

[B15] Backer S. Hidalgo-Sanchez M. Offner N. Portales-Casamar E. Debant A. Fort P. (2007). Trio controls the mature organization of neuronal clusters in the hindbrain. J. Neurosci. 27, 10323–10332 10.1523/JNEUROSCI.1102-07.2007 17898204 PMC6673147

[B16] Backer S. Sakurai T. Grumet M. Sotelo C. Bloch-Gallego E. (2002). Nr-CAM and TAG-1 are expressed in distinct populations of developing precerebellar and cerebellar neurons. Neuroscience 113, 743–748 10.1016/S0306-4522(02)00221-X 12182881

[B17] Belluardo N. Mudo G. Trovato-Salinaro A. Le Gurun S. Charollais A. Serre-Beinier V. (2000). Expression of connexin36 in the adult and developing rat brain. Brain Res. 865, 121–138 10.1016/S0006-8993(00)02300-3 10814742

[B18] Blenkinsop T. A. Lang E. J. (2006). Block of inferior olive gap junctional coupling decreases Purkinje cell complex spike synchrony and rhythmicity. J. Neurosci. 26, 1739–1748 10.1523/JNEUROSCI.3677-05.2006 16467522 PMC6793617

[B19] Bloch-Gallego E. Ezan F. Tessier-Lavigne M. Sotelo C. (1999). Floor plate and netrin-1 are involved in the migration and survival of inferior olivary neurons. J. Neurosci. 19, 4407–4420 10341242 10.1523/JNEUROSCI.19-11-04407.1999 PMC6782586

[B20] Bosman L. W. Takechi H. Hartmann J. Eilers J. Konnerth A. (2008). Homosynaptic long-term synaptic potentiation of the “winner” climbing fiber synapse in developing Purkinje cells. J. Neurosci. 28, 798–807 10.1523/JNEUROSCI.4074-07.2008 18216188 PMC6671003

[B21] Bostan A. C. Dum R. P. Strick P. L. (2010). The basal ganglia communicate with the cerebellum. Proc. Natl. Acad. Sci. U.S.A. 107, 8452–8456 10.1073/pnas.1000496107 20404184 PMC2889518

[B22] Bourrat F. Sotelo C. (1988). Migratory pathways and neuritic differentiation of inferior olivary neurons in the rat embryo. Axonal tracing study using the *in vitro* slab technique. Brain Res. 467, 19–37 3359328 10.1016/0165-3806(88)90064-8

[B23] Bourrat F. Sotelo C. (1990a). Early development of the rat precerebellar system: migratory routes, selective aggregation and neuritic differentiation of the inferior olive and lateral reticular nucleus neurons. An overview. Arch. Ital. Biol. 128, 151–170 2268181

[B24] Bourrat F. Sotelo C. (1990b). Migratory pathways and selective aggregation of the lateral reticular neurons in the rat embryo: a horseradish peroxidase *in vitro* study, with special reference to migration patterns of the precerebellar nuclei. J. Comp. Neurol. 294, 1–13 10.1002/cne.902940102 2324326

[B25] Bourrat F. Sotelo C. (1991). Relationships between neuronal birthdates and cytoarchitecture in the rat inferior olivary complex. J. Comp. Neurol. 313, 509–521 10.1002/cne.903130311 1770173

[B26] Boyden E. S. Zhang F. Bamberg E. Nagel G. Deisseroth K. (2005). Millisecond-timescale, genetically targeted optical control of neural activity. Nat. Neurosci. 8, 1263–1268 10.1038/nn1525 16116447

[B27] Bozza T. Feinstein P. Zheng C. Mombaerts P. (2002). Odorant receptor expression defines functional units in the mouse olfactory system. J. Neurosci. 22, 3033–3043 11943806 10.1523/JNEUROSCI.22-08-03033.2002 PMC6757547

[B28] Bradley D. J. Ghelarducci B. Spyer K. M. (1991). The role of the posterior cerebellar vermis in cardiovascular control. Neurosci. Res. 12, 45–56 10.1016/0168-0102(91)90099-K 1660994

[B29] Bravin M. Rossi F. Strata P. (1995). Different climbing fibres innervate separate dendritic regions of the same Purkinje cell in hypogranular cerebellum. J. Comp. Neurol. 357, 395–407 10.1002/cne.903570306 7673475

[B30] Brochu G. Maler L. Hawkes R. (1990). Zebrin II: a polypeptide antigen expressed selectively by Purkinje cells reveals compartments in rat and fish cerebellum. J. Comp. Neurol. 291, 538–552 10.1002/cne.902910405 2329190

[B31] Cajal R. Y. (1911). Histologie du Systeme Nerveux de l'homme et des Vertebres. Vol. II Paris: Maloine

[B32] Callaway E. M. (2008). Transneuronal circuit tracing with neurotropic viruses. Curr. Opin. Neurobiol. 18, 617–623 10.1016/j.conb.2009.03.007 19349161 PMC2698966

[B33] Cang J. Niell C. M. Liu X. Pfeiffenberger C. Feldheim D. A. Stryker M. P. (2008a). Selective disruption of one Cartesian axis of cortical maps and receptive fields by deficiency in ephrin-As and structured activity. Neuron 57, 511–523 10.1016/j.neuron.2007.12.025 18304481 PMC2413327

[B34] Cang J. Wang L. Stryker M. P. Feldheim D. A. (2008b). Roles of ephrin-as and structured activity in the development of functional maps in the superior colliculus. J. Neurosci. 28, 11015–11023 10.1523/JNEUROSCI.2478-08.2008 18945909 PMC2588436

[B35] Causeret F. Danne F. Ezan F. Sotelo C. Bloch-Gallego E. (2002). Slit antagonizes netrin-1 attractive effects during the migration of inferior olivary neurons. Dev. Biol. 246, 429–440 10.1006/dbio.2002.0681 12051827

[B36] Cerminara N. L. (2010). Cerebellar modules: individual or composite entities? J. Neurosci. 30, 16065–16067 10.1523/JNEUROSCI.4823-10.2010 21123553 PMC6634852

[B37] Cerminara N. L. Apps R. (2011). Behavioural significance of cerebellar modules. Cerebellum 10, 484–494 10.1007/s12311-010-0209-2 20838949 PMC3169775

[B38] Chedotal A. Pourquie O. Ezan F. San Clemente H. Sotelo C. (1996). BEN as a presumptive target recognition molecule during the development of the olivocerebellar system. J. Neurosci. 16, 3296–3310 8627367 10.1523/JNEUROSCI.16-10-03296.1996 PMC6579157

[B39] Chedotal A. Sotelo C. (1992). Early development of olivocerebellar projections in the fetal rat using CGRP immunocytochemistry. Eur. J. Neurosci. 4, 1159–1179 12106421 10.1111/j.1460-9568.1992.tb00142.x

[B40] Chedotal A. Sotelo C. (1993). The ‘creeper stage’ in cerebellar climbing fiber synaptogenesis precedes the ‘pericellular nest’–ultrastructural evidence with parvalbumin immunocytochemistry. Brain Res. Dev. Brain Res. 76, 207–220 8149587 10.1016/0165-3806(93)90209-s

[B41] Chen L. Bao S. Lockard J. M. Kim J. K. Thompson R. F. (1996). Impaired classical eyeblink conditioning in cerebellar-lesioned and Purkinje cell degeneration (pcd) mutant mice. J. Neurosci. 16, 2829–2838 8786457 10.1523/JNEUROSCI.16-08-02829.1996 PMC6578747

[B42] Chockkan V. Hawkes R. (1994). Functional and antigenic maps in the rat cerebellum: zebrin compartmentation and vibrissal receptive fields in lobule IXa. J. Comp. Neurol. 345, 33–45 10.1002/cne.903450103 8089276

[B43] Coffman K. A. Dum R. P. Strick P. L. (2011). Cerebellar vermis is a target of projections from the motor areas in the cerebral cortex. Proc. Natl. Acad. Sci. U.S.A. 108, 16068–16073 10.1073/pnas.1107904108 21911381 PMC3179064

[B44] Coulon P. Derbin C. Kucera P. Lafay F. Prehaud C. Flamand A. (1989). Invasion of the peripheral nervous systems of adult mice by the CVS strain of rabies virus and its avirulent derivative AvO1. J. Virol. 63, 3550–3554 2664219 10.1128/jvi.63.8.3550-3554.1989 PMC250937

[B45] Crepel F. Delhaye-Bouchaud N. (1979). Distribution of climbing fibres on cerebellar Purkinje cells in X-irradiated rats. An electrophysiological study. J. Physiol. 290, 97–112 469809 10.1113/jphysiol.1979.sp012762 PMC1278826

[B46] Crepel F. Delhaye-Bouchaud N. Guastavino J. M. Sampaio I. (1980). Multiple innervation of cerebellar Purkinje cells by climbing fibres in staggerer mutant mouse. Nature 283, 483–484 7352029 10.1038/283483a0

[B47] Crepel F. Mariani J. (1976). Multiple innervation of Purkinje cells by climbing fibers in the cerebellum of the weaver mutant mouse. J. Neurobiol. 7, 579–582 10.1002/neu.480070610 1003203

[B48] Crepel F. Mariani J. Delhaye-Bouchaud N. (1976). Evidence for a multiple innervation of Purkinje cells by climbing fibers in the immature rat cerebellum. J. Neurobiol. 7, 567–578 10.1002/neu.480070609 1003202

[B49] Cummings S. L. Young W. S. 3rd. Bishop G. A. De Souza E. B. King J. S. (1989). Distribution of corticotropin-releasing factor in the cerebellum and precerebellar nuclei of the opossum: a study utilizing immunohistochemistry, *in situ* hybridization histochemistry, and receptor autoradiography. J. Comp. Neurol. 280, 501–521 10.1002/cne.902800402 2785124

[B50] de Diego I. Kyriakopoulou K. Karagogeos D. Wassef M. (2002). Multiple influences on the migration of precerebellar neurons in the caudal medulla. Development 129, 297–306 11807023 10.1242/dev.129.2.297

[B51] Degen J. Meier C. Van Der Giessen R. S. Sohl G. Petrasch-Parwez E. Urschel S. (2004). Expression pattern of lacZ reporter gene representing connexin36 in transgenic mice. J. Comp. Neurol. 473, 511–525 10.1002/cne.20085 15116387

[B52] Dehnes Y. Chaudhry F. A. Ullensvang K. Lehre K. P. Storm-Mathisen J. Danbolt N. C. (1998). The glutamate transporter EAAT4 in rat cerebellar Purkinje cells: a glutamate-gated chloride channel concentrated near the synapse in parts of the dendritic membrane facing astroglia. J. Neurosci. 18, 3606–3619 9570792 10.1523/JNEUROSCI.18-10-03606.1998 PMC6793133

[B53] Deisseroth K. Feng G. Majewska A. K. Miesenbock G. Ting A. Schnitzer M. J. (2006). Next-generation optical technologies for illuminating genetically targeted brain circuits. J. Neurosci. 26, 10380–10386 10.1523/JNEUROSCI.3863-06.2006 17035522 PMC2820367

[B54] Demilly A. Reeber S. L. Gebre S. A. Sillitoe R. V. (2011). Neurofilament heavy chain expression reveals a unique parasagittal stripe topography in the mouse cerebellum. Cerebellum 10, 409–421 10.1007/s12311-010-0156-y 20127431

[B55] Dixon K. J. Hilber W. Speare S. Willson M. L. Bower A. J. Sherrard R. M. (2005). Post-lesion transcommissural olivocerebellar reinnervation improves motor function following unilateral pedunculotomy in the neonatal rat. Exp. Neurol. 196, 254–265 10.1016/j.expneurol.2005.07.018 16125176

[B56] Dixon K. J. Sherrard R. M. (2006). Brain-derived neurotrophic factor induces post-lesion transcommissural growth of olivary axons that develop normal climbing fibers on mature Purkinje cells. Exp. Neurol. 202, 44–56 10.1016/j.expneurol.2006.05.010 16790241

[B57] Ebner T. J. Wang X. Gao W. Cramer S. W. Chen G. (2012). Parasagittal zones in the cerebellar cortex differ in excitability, information processing, and synaptic plasticity. Cerebellum 11, 418–419 10.1007/s12311-011-0347-1 22249913 PMC3856581

[B58] Ekerot C. F. Larson B. (1980). Termination in overlapping sagittal zones in cerebellar anterior lobe of mossy and climbing fiber paths activated from dorsal funiculus. Exp. Brain Res. 38, 163–172 7358102 10.1007/BF00236737

[B59] Errante L. Tang D. Gardon M. Sekerkova G. Mugnaini E. Shaw G. (1998). The intermediate filament protein peripherin is a marker for cerebellar climbing fibres. J. Neurocytol. 27, 69–84 9609398 10.1023/a:1006991104595

[B60] Flanagan J. G. Vanderhaeghen P. (1998). The ephrins and Eph receptors in neural development. Annu. Rev. Neurosci. 21, 309–345 10.1146/annurev.neuro.21.1.309 9530499

[B61] Friedman G. C. O'Leary D. D. (1996). Retroviral misexpression of engrailed genes in the chick optic tectum perturbs the topographic targeting of retinal axons. J. Neurosci. 16, 5498–5509 8757262 10.1523/JNEUROSCI.16-17-05498.1996 PMC6578875

[B62] Fujita H. Oh-Nishi A. Obayashi S. Sugihara I. (2010). Organization of the marmoset cerebellum in three-dimensional space: lobulation, aldolase C compartmentalization and axonal projection. J. Comp. Neurol. 518, 1764–1791 10.1002/cne.22301 20235174

[B63] Graf W. Gerrits N. Yatim-Dhiba N. Ugolini G. (2002). Mapping the oculomotor system: the power of transneuronal labelling with rabies virus. Eur. J. Neurosci. 15, 1557–1562 10.1046/j.1460-9568.2002.01994.x 12028367

[B64] Graham D. J. Wylie D. R. (2012). Zebrin-immunopositive and -immunonegative stripe pairs represent functional units in the pigeon vestibulocerebellum. J. Neurosci. 32, 12769–12779 10.1523/JNEUROSCI.0197-12.2012 22973000 PMC6703799

[B65] Hallem J. S. Thompson J. H. Gundappa-Sulur G. Hawkes R. Bjaalie J. G. Bower J. M. (1999). Spatial correspondence between tactile projection patterns and the distribution of the antigenic Purkinje cell markers anti-zebrin I and anti-zebrin II in the cerebellar folium crus IIA of the rat. Neuroscience 93, 1083–1094 10.1016/S0306-4522(99)00144-X 10473273

[B66] Hashimoto K. Ichikawa R. Kitamura K. Watanabe M. Kano M. (2009). Translocation of a “winner” climbing fiber to the Purkinje cell dendrite and subsequent elimination of “losers” from the soma in developing cerebellum. Neuron 63, 106–118 10.1016/j.neuron.2009.06.008 19607796

[B67] Hashimoto K. Kano M. (2003). Functional differentiation of multiple climbing fiber inputs during synapse elimination in the developing cerebellum. Neuron 38, 785–796 10.1016/S0896-6273(03)00298-8 12797962

[B68] Hashimoto K. Kano M. (2005). Postnatal development and synapse elimination of climbing fiber to Purkinje cell projection in the cerebellum. Neurosci. Res. 53, 221–228 10.1016/j.neures.2005.07.007 16139911

[B69] Hashimoto K. Tsujita M. Miyazaki T. Kitamura K. Yamazaki M. Shin H. S. (2011). Postsynaptic P/Q-type Ca2+ channel in Purkinje cell mediates synaptic competition and elimination in developing cerebellum. Proc. Natl. Acad. Sci. U.S.A. 108, 9987–9992 10.1073/pnas.1101488108 21628556 PMC3116426

[B70] Hashimoto M. Hibi M. (2012). Development and evolution of cerebellar neural circuits. Dev. Growth Differ. 54, 373–389 10.1111/j.1440-169X.2012.01348.x 22524607

[B71] Hashimoto M. Ito R. Kitamura N. Namba K. Hisano Y. (2012). Epha4 controls the midline crossing and contralateral axonal projections of inferior olive neurons. J. Comp. Neurol. 520, 1702–1720 10.1002/cne.23008 22121026

[B72] Hashimoto M. Mikoshiba K. (2003). Mediolateral compartmentalization of the cerebellum is determined on the “birth date” of Purkinje cells. J. Neurosci. 23, 11342–11351 14672998 10.1523/JNEUROSCI.23-36-11342.2003 PMC6740522

[B73] Hawkes R. Colonnier M. Leclerc N. (1985). Monoclonal antibodies reveal sagittal banding in the rodent cerebellar cortex. Brain Res. 333, 359–365 10.1016/0006-8993(85)91593-8 3888348

[B74] Hawkes R. Herrup K. (1995). Aldolase C/zebrin II and the regionalization of the cerebellum. J. Mol. Neurosci. 6, 147–158 10.1007/BF02736761 8672398

[B75] Hawkes R. Leclerc N. (1987). Antigenic map of the rat cerebellar cortex: the distribution of parasagittal bands as revealed by monoclonal anti-Purkinje cell antibody mabQ113. J. Comp. Neurol. 256, 29–41 10.1002/cne.902560104 3546410

[B76] Hidalgo-Sanchez M. Backer S. Puelles L. Bloch-Gallego E. (2012). Origin and plasticity of the subdivisions of the inferior olivary complex. Dev. Biol. 371, 215–226 10.1016/j.ydbio.2012.08.019 22960232

[B77] Hira R. Honkura N. Noguchi J. Maruyama Y. Augustine G. J. Kasai H. (2009). Transcranial optogenetic stimulation for functional mapping of the motor cortex. J. Neurosci. Methods 179, 258–263 10.1016/j.jneumeth.2009.02.001 19428535

[B78] Hirai H. Pang Z. Bao D. Miyazaki T. Li L. Miura E. (2005). Cbln1 is essential for synaptic integrity and plasticity in the cerebellum. Nat. Neurosci. 8, 1534–1541 10.1038/nn1576 16234806

[B79] Hisano S. Sawada K. Kawano M. Kanemoto M. Xiong G. Mogi K. (2002). Expression of inorganic phosphate/vesicular glutamate transporters (BNPI/VGLUT1 and DNPI/VGLUT2) in the cerebellum and precerebellar nuclei of the rat. Brain Res. Mol. Brain Res. 107, 23–31 10.1016/S0169-328X(02)00442-4 12414120

[B80] Horn K. M. Pong M. Gibson A. R. (2010). Functional relations of cerebellar modules of the cat. J. Neurosci. 30, 9411–9423 10.1523/JNEUROSCI.0440-10.2010 20631170 PMC3865504

[B81] Hoshino M. Nakamura S. Mori K. Kawauchi T. Terao M. Nishimura Y. V. (2005). Ptf1a, a bHLH transcriptional gene, defines GABAergic neuronal fates in cerebellum. Neuron 47, 201–213 10.1016/j.neuron.2005.06.007 16039563

[B82] Hubel D. H. Wiesel T. N. (1979). Brain mechanisms of vision. Sci. Am. 241, 150–162 91195 10.1038/scientificamerican0979-150

[B83] Huffman K. J. Cramer K. S. (2007). EphA4 misexpression alters tonotopic projections in the auditory brainstem. Dev. Neurobiol. 67, 1655–1668 10.1002/dneu.20535 17577206

[B84] Ichikawa R. Miyazaki T. Kano M. Hashikawa T. Tatsumi H. Sakimura K. (2002). Distal extension of climbing fiber territory and multiple innervation caused by aberrant wiring to adjacent spiny branchlets in cerebellar Purkinje cells lacking glutamate receptor delta 2. J. Neurosci. 22, 8487–8503 12351723 10.1523/JNEUROSCI.22-19-08487.2002 PMC6757771

[B85] Jaarsma D. Ruigrok T. J. Caffe R. Cozzari C. Levey A. I. Mugnaini E. (1997). Cholinergic innervation and receptors in the cerebellum. Prog. Brain Res. 114, 67–96 9193139 10.1016/s0079-6123(08)63359-2

[B86] Johnston A. (1989). The geometry of the topographic map in striate cortex. Vision Res. 29, 1493–1500 2635475 10.1016/0042-6989(89)90133-8

[B87] Kano M. Hashimoto K. (2009). Synapse elimination in the central nervous system. Curr. Opin. Neurobiol. 19, 154–161 10.1016/j.conb.2009.05.002 19481442

[B88] Kano M. Hashimoto K. Chen C. Abeliovich A. Aiba A. Kurihara H. (1995). Impaired synapse elimination during cerebellar development in PKC gamma mutant mice. Cell 83, 1223–1231 10.1016/0092-8674(95)90147-7 8548808

[B89] Kano M. Hashimoto K. Kurihara H. Watanabe M. Inoue Y. Aiba A. (1997). Persistent multiple climbing fiber innervation of cerebellar Purkinje cells in mice lacking mGluR1. Neuron 18, 71–79 10.1016/S0896-6273(01)80047-7 9010206

[B90] Kano M. Hashimoto K. Watanabe M. Kurihara H. Offermanns S. Jiang H. (1998). Phospholipase cbeta4 is specifically involved in climbing fiber synapse elimination in the developing cerebellum. Proc. Natl. Acad. Sci. U.S.A. 95, 15724–15729 10.1073/pnas.95.26.15724 9861037 PMC28111

[B91] Karam S. D. Burrows R. C. Logan C. Koblar S. Pasquale E. B. Bothwell M. (2000). Eph receptors and ephrins in the developing chick cerebellum: relationship to sagittal patterning and granule cell migration. J. Neurosci. 20, 6488–6500 10964955 10.1523/JNEUROSCI.20-17-06488.2000 PMC6772988

[B92] Karam S. D. Dottori M. Ogawa K. Henderson J. T. Boyd A. W. Pasquale E. B. (2002). EphA4 is not required for Purkinje cell compartmentation. Brain Res. Dev. Brain Res. 135, 29–38 10.1016/S0165-3806(02)00278-X 11978390

[B93] Kashiwabuchi N. Ikeda K. Araki K. Hirano T. Shibuki K. Takayama C. (1995). Impairment of motor coordination, Purkinje cell synapse formation, and cerebellar long-term depression in GluR delta 2 mutant mice. Cell 81, 245–252 10.1016/0092-8674(95)90334-8 7736576

[B94] Kelly R. M. Strick P. L. (2000). Rabies as a transneuronal tracer of circuits in the central nervous system. J. Neurosci. Methods 103, 63–71 10.1016/S0165-0270(00)00296-X 11074096

[B95] Kelly R. M. Strick P. L. (2003). Cerebellar loops with motor cortex and prefrontal cortex of a nonhuman primate. J. Neurosci. 23, 8432–8444 12968006 10.1523/JNEUROSCI.23-23-08432.2003 PMC6740694

[B96] Landsberg R. L. Awatramani R. B. Hunter N. L. Farago A. F. DiPietrantonio H. J. Rodriguez C. I. (2005). Hindbrain rhombic lip is comprised of discrete progenitor cell populations allocated by Pax6. Neuron 48, 933–947 10.1016/j.neuron.2005.11.031 16364898

[B97] Lang E. J. Sugihara I. Welsh J. P. Llinas R. (1999). Patterns of spontaneous purkinje cell complex spike activity in the awake rat. J. Neurosci. 19, 2728–2739 10087085 10.1523/JNEUROSCI.19-07-02728.1999 PMC6786059

[B98] Larouche M. Che P. M. Hawkes R. (2006). Neurogranin expression identifies a novel array of Purkinje cell parasagittal stripes during mouse cerebellar development. J. Comp. Neurol. 494, 215–227 10.1002/cne.20791 16320235

[B99] Larsell O. (1952). The morphogenesis and adult pattern of the lobules and fissures of the cerebellum of the white rat. J. Comp. Neurol. 97, 281–356 12999992 10.1002/cne.900970204

[B100] Leergaard T. B. Bjaalie J. G. (2007). Topography of the complete corticopontine projection: from experiments to principal Maps. Front. Neurosci. 1:1 10.3389/neuro.01.1.1.016.2007 18982130 PMC2518056

[B101] Li H. Crair M. C. (2011). How do barrels form in somatosensory cortex? Ann. N.Y. Acad. Sci. 1225, 119–129 10.1111/j.1749-6632.2011.06024.x 21534999 PMC4700879

[B102] Llinas R. Sasaki K. (1989). The functional organization of the olivo-cerebellar system as examined by multiple Purkinje cell recordings. Eur. J. Neurosci. 1, 587–602 12106117 10.1111/j.1460-9568.1989.tb00365.x

[B103] Llinas R. Walton K. Hillman D. E. Sotelo C. (1975). Inferior olive: its role in motor learing. Science 190, 1230–1231 10.1126/science.128123 128123

[B104] Logan C. Wizenmann A. Drescher U. Monschau B. Bonhoeffer F. Lumsden A. (1996). Rostral optic tectum acquires caudal characteristics following ectopic engrailed expression. Curr. Biol. 6, 1006–1014 10.1016/S0960-9822(02)00645-0 8805331

[B105] Machold R. Fishell G. (2005). Math1 is expressed in temporally discrete pools of cerebellar rhombic-lip neural progenitors. Neuron 48, 17–24 10.1016/j.neuron.2005.08.028 16202705

[B4] Madisen L. Zwingman T. A. Sunkin S. M. Wook Oh S. Zariwala H. A. Gu H. (2010). A robust and high-throughput Cre reporting and characterization system for the whole mouse brain. Nat. Neurosci. 13, 133–140 10.1038/nn.2467 20023653 PMC2840225

[B106] Maklad A. Fritzsch B. (2003). Partial segregation of posterior crista and saccular fibers to the nodulus and uvula of the cerebellum in mice, and its development. Brain Res. Dev. Brain Res. 140, 223–236 10.1016/S0165-3806(02)00609-0 12586428

[B107] Mariani J. Changeux J. P. (1980). Multiple innervation of Purkinje cells by climbing fibers in the cerebellum of the adult staggerer mutant mouse. J. Neurobiol. 11, 41–50 10.1002/neu.480110106 6243692

[B108] Mariani J. Changeux J. P. (1981). Ontogenesis of olivocerebellar relationships. I. Studies by intracellular recordings of the multiple innervation of Purkinje cells by climbing fibers in the developing rat cerebellum. J. Neurosci. 1, 696–702 7346578 10.1523/JNEUROSCI.01-07-00696.1981 PMC6564201

[B109] Mariani J. Crepel F. Mikoshiba K. Changeux J. P. Sotelo C. (1977). Anatomical, physiological and biochemical studies of the cerebellum from Reeler mutant mouse. Philos. Trans. R. Soc. Lond. B Biol. Sci. 281, 1–28 10.1098/rstb.1977.0121 22882

[B110] Marillat V. Sabatier C. Failli V. Matsunaga E. Sotelo C. Tessier-Lavigne M. (2004). The slit receptor Rig-1/Robo3 controls midline crossing by hindbrain precerebellar neurons and axons. Neuron 43, 69–79 10.1016/j.neuron.2004.06.018 15233918

[B111] Marshel J. H. Mori T. Nielsen K. J. Callaway E. M. (2010). Targeting single neuronal networks for gene expression and cell labeling *in vivo*. Neuron 67, 562–574 10.1016/j.neuron.2010.08.001 20797534 PMC2929426

[B112] Martin E. I. Ressler K. J. Jasnow A. M. Dabrowska J. Hazra R. Rainnie D. G. (2010). A novel transgenic mouse for gene-targeting within cells that express corticotropin-releasing factor. Biol. Psychiatry 67, 1212–1216 10.1016/j.biopsych.2010.01.026 20303068 PMC3039842

[B113] Mason C. A. Christakos S. Catalano S. M. (1990). Early climbing fiber interactions with Purkinje cells in the postnatal mouse cerebellum. J. Comp. Neurol. 297, 77–90 10.1002/cne.902970106 1695909

[B114] Mason C. A. Gregory E. (1984). Postnatal maturation of cerebellar mossy and climbing fibers: transient expression of dual features on single axons. J. Neurosci. 4, 1715–1735 6737039 10.1523/JNEUROSCI.04-07-01715.1984 PMC6564888

[B115] McCormick D. A. Thompson R. F. (1984). Cerebellum: essential involvement in the classically conditioned eyelid response. Science 223, 296–299 10.1126/science.6701513 6701513

[B116] Miyazaki T. Hashimoto K. Shin H. S. Kano M. Watanabe M. (2004). P/Q-type Ca2+ channel alpha1A regulates synaptic competition on developing cerebellar Purkinje cells. J. Neurosci. 24, 1734–1743 10.1523/JNEUROSCI.4208-03.2004 14973254 PMC6730452

[B117] Miyazaki T. Yamasaki M. Takeuchi T. Sakimura K. Mishina M. Watanabe M. (2010). Ablation of glutamate receptor GluRdelta2 in adult Purkinje cells causes multiple innervation of climbing fibers by inducing aberrant invasion to parallel fiber innervation territory. J. Neurosci. 30, 15196–15209 10.1523/JNEUROSCI.0934-10.2010 21068325 PMC6633829

[B118] Morara S. Marcotti W. Provini L. Rosina A. (1997). Neuropeptide, Y (NPY) expression is up-regulated in the rat inferior olive during development. Neuroreport 8, 3743–3747 9427362 10.1097/00001756-199712010-00017

[B119] Morara S. Rosina A. Provini L. (1992). CGRP as a marker of the climbing fibers during the development of the cerebellum in the rat. Ann. N.Y. Acad. Sci. 657, 461–463 10.1111/j.1749-6632.1992.tb22800.x 1637100

[B120] Nakayama H. Miyazaki T. Kitamura K. Hashimoto K. Yanagawa Y. Obata K. (2012). GABAergic inhibition regulates developmental synapse elimination in the cerebellum. Neuron 74, 384–396 10.1016/j.neuron.2012.02.032 22542190

[B121] Namba K. Sugihara I. Hashimoto M. (2011). Close correlation between the birth date of Purkinje cells and the longitudinal compartmentalization of the mouse adult cerebellum. J. Comp. Neurol. 519, 2594–2614 10.1002/cne.22640 21456012

[B122] Neudert F. Nuernberger K. K. Redies C. (2008). Comparative analysis of cadherin expression and connectivity patterns in the cerebellar system of ferret and mouse. J. Comp. Neurol. 511, 736–752 10.1002/cne.21865 18855899

[B123] Nicholls J. Saunders N. (1996). Regeneration of immature mammalian spinal cord after injury. Trends Neurosci. 19, 229–234 10.1016/0166-2236(96)10021-7 8761958

[B124] Nichols D. H. Bruce L. L. (2006). Migratory routes and fates of cells transcribing the Wnt-1 gene in the murine hindbrain. Dev. Dyn. 235, 285–300 10.1002/dvdy.20611 16273520

[B125] Nishida K. Flanagan J. G. Nakamoto M. (2002). Domain-specific olivocerebellar projection regulated by the EphA-ephrin-A interaction. Development 129, 5647–5658 10.1242/dev.00162 12421705

[B126] Nishiyama H. Fukaya M. Watanabe M. Linden D. J. (2007). Axonal motility and its modulation by activity are branch-type specific in the intact adult cerebellum. Neuron 56, 472–487 10.1016/j.neuron.2007.09.010 17988631 PMC2098835

[B127] O'Hearn E. Long D. B. Molliver M. E. (1993). Ibogaine induces glial activation in parasagittal zones of the cerebellum. Neuroreport 4, 299–302 8477052 10.1097/00001756-199303000-00018

[B128] O'Hearn E. Molliver M. E. (1993). Degeneration of Purkinje cells in parasagittal zones of the cerebellar vermis after treatment with ibogaine or harmaline. Neuroscience 55, 303–310 10.1016/0306-4522(93)90500-F 8377927

[B129] O'Leary J. L. Inukai J. Smith J. M. (1971). Histogenesis of the cerebellar climbing fiber in the rat. J. Comp. Neurol. 142, 377–391 10.1002/cne.901420307 5566083

[B130] Oberdick J. Smeyne R. J. Mann J. R. Zackson S. Morgan J. I. (1990). A promoter that drives transgene expression in cerebellar Purkinje and retinal bipolar neurons. Science 248, 223–226 10.1126/science.2109351 2109351

[B131] Offermanns S. Hashimoto K. Watanabe M. Sun W. Kurihara H. Thompson R. F. (1997). Impaired motor coordination and persistent multiple climbing fiber innervation of cerebellar Purkinje cells in mice lacking Galphaq. Proc. Natl. Acad. Sci. U.S.A. 94, 14089–14094 9391157 10.1073/pnas.94.25.14089 PMC28437

[B132] Ozol K. Hayden J. M. Oberdick J. Hawkes R. (1999). Transverse zones in the vermis of the mouse cerebellum. J. Comp. Neurol. 412, 95–111 10.1002/(SICI)1096-9861(19990913)412:1<95::AID-CNE7>3.0.CO;2-Y 10440712

[B133] Pakan J. M. Wylie D. R. (2008). Congruence of zebrin II expression and functional zones defined by climbing fiber topography in the flocculus. Neuroscience 157, 57–69 10.1016/j.neuroscience.2008.08.062 18824220

[B134] Paradies M. A. Eisenman L. M. (1993). Evidence of early topographic organization in the embryonic olivocerebellar projection: a model system for the study of pattern formation processes in the central nervous system. Dev. Dyn. 197, 125–145 10.1002/aja.1001970206 8219355

[B135] Paradies M. A. Grishkat H. Smeyne R. J. Oberdick J. Morgan J. I. Eisenman L. M. (1996). Correspondence between L7-lacZ-expressing Purkinje cells and labeled olivocerebellar fibers during late embryogenesis in the mouse. J. Comp. Neurol. 374, 451–466 10.1002/(SICI)1096-9861(19961021)374:3<451::AID-CNE9>3.0.CO;2-0 8906510

[B136] Pascual M. Abasolo I. Mingorance-Le Meur A. Martinez A. Del Rio J. A. Wright C. V. (2007). Cerebellar GABAergic progenitors adopt an external granule cell-like phenotype in the absence of Ptf1a transcription factor expression. Proc. Natl. Acad. Sci. U.S.A. 104, 5193–5198 10.1073/pnas.0605699104 17360405 PMC1829285

[B137] Paukert M. Huang Y. H. Tanaka K. Rothstein J. D. Bergles D. E. (2010). Zones of enhanced glutamate release from climbing fibers in the mammalian cerebellum. J. Neurosci. 30, 7290–7299 10.1523/JNEUROSCI.5118-09.2010 20505095 PMC2894469

[B138] Pierce E. T. (1973). Time of origin of neurons in the brain stem of the mouse. Prog. Brain Res. 40, 53–65 10.1016/S0079-6123(08)60679-2 4802670

[B139] Pijpers A. Winkelman B. H. Bronsing R. Ruigrok T. J. (2008). Selective impairment of the cerebellar C1 module involved in rat hind limb control reduces step-dependent modulation of cutaneous reflexes. J. Neurosci. 28, 2179–2189 10.1523/JNEUROSCI.4668-07.2008 18305251 PMC6671855

[B140] Rancz E. A. Franks K. M. Schwarz M. K. Pichler B. Schaefer A. T. Margrie T. W. (2011). Transfection via whole-cell recording *in vivo*: bridging single-cell physiology, genetics and connectomics. Nat. Neurosci. 14, 527–532 10.1038/nn.2765 21336272 PMC3501644

[B141] Redies C. Neudert F. Lin J. (2011). Cadherins in cerebellar development: translation of embryonic patterning into mature functional compartmentalization. Cerebellum 10, 393–408 10.1007/s12311-010-0207-4 20820976

[B142] Reeber S. L. Sillitoe R. V. (2011). Patterned expression of a cocaine- and amphetamine-regulated transcript peptide reveals complex circuit topography in the rodent cerebellar cortex. J. Comp. Neurol. 519, 1781–1796 10.1002/cne.22601 21452228

[B143] Rekling J. C. Jensen K. H. Jahnsen H. (2012). Spontaneous cluster activity in the inferior olivary nucleus in brainstem slices from postnatal mice. J. Physiol. 590, 1547–1562 10.1113/jphysiol.2011.222570 22250213 PMC3413494

[B144] Rodriguez C. I. Dymecki S. M. (2000). Origin of the precerebellar system. Neuron 27, 475–486 10.1016/S0896-6273(00)00059-3 11055431

[B145] Ruigrok T. J. (2011). Ins and outs of cerebellar modules. Cerebellum 10, 464–474 10.1007/s12311-010-0164-y 20232190 PMC3169761

[B146] Sacchetti B. Scelfo B. Strata P. (2009). Cerebellum and emotional behavior. Neuroscience 162, 756–762 10.1016/j.neuroscience.2009.01.064 19409218

[B147] Sarna J. R. Marzban H. Watanabe M. Hawkes R. (2006). Complementary stripes of phospholipase Cbeta3 and Cbeta4 expression by Purkinje cell subsets in the mouse cerebellum. J. Comp. Neurol. 496, 303–313 10.1002/cne.20912 16566000

[B148] Sasaki K. Bower J. M. Llinas R. (1989). Multiple Purkinje cell recording in rodent cerebellar cortex. Eur. J. Neurosci. 1, 572–586 12106116 10.1111/j.1460-9568.1989.tb00364.x

[B149] Sawada K. Fukui Y. Hawkes R. (2008). Spatial distribution of corticotropin-releasing factor immunopositive climbing fibers in the mouse cerebellum: analysis by whole mount immunohistochemistry. Brain Res. 1222, 106–117 10.1016/j.brainres.2008.05.029 18572150

[B150] Sawada K. Sakata-Haga H. Hisano S. Fukui Y. (2001). Topological relationship between corticotropin-releasing factor-immunoreactive cerebellar afferents and tyrosine hydroxylase-immunoreactive Purkinje cells in a hereditary ataxic mutant, rolling mouse Nagoya. Neuroscience 102, 925–935 10.1016/S0306-4522(00)00533-9 11182254

[B151] Seoane A. Apps R. Balbuena E. Herrero L. Llorens J. (2005). Differential effects of trans-crotononitrile and 3-acetylpyridine on inferior olive integrity and behavioural performance in the rat. Eur. J. Neurosci. 22, 880–894 10.1111/j.1460-9568.2005.04230.x 16115211

[B152] Sherrard R. M. Bower A. J. Payne J. N. (1986). Innervation of the adult rat cerebellar hemisphere by fibres from the ipsilateral inferior olive following unilateral neonatal pedunculotomy: an autoradiographic and retrograde fluorescent double-labelling study. Exp. Brain Res. 62, 411–421 3709723 10.1007/BF00238860

[B153] Sillitoe R. V. Gopal N. Joyner A. L. (2009). Embryonic origins of ZebrinII parasagittal stripes and establishment of topographic Purkinje cell projections. Neuroscience 162, 574–588 10.1016/j.neuroscience.2008.12.025 19150487 PMC2716412

[B154] Sillitoe R. V. Hawkes R. (2002). Whole-mount immunohistochemistry: a high-throughput screen for patterning defects in the mouse cerebellum. J. Histochem. Cytochem. 50, 235–244 10.1177/002215540205000211 11799142

[B155] Sillitoe R. V. Joyner A. L. (2007). Morphology, molecular codes, and circuitry produce the three-dimensional complexity of the cerebellum. Annu. Rev. Cell Dev. Biol. 23, 549–577 10.1146/annurev.cellbio.23.090506.123237 17506688

[B156] Sillitoe R. V. Marzban H. Larouche M. Zahedi S. Affanni J. Hawkes R. (2005). Conservation of the architecture of the anterior lobe vermis of the cerebellum across mammalian species. Prog. Brain Res. 148, 283–297 10.1016/S0079-6123(04)48022-4 15661197

[B157] Sillitoe R. V. Vogel M. W. Joyner A. L. (2010). Engrailed homeobox genes regulate establishment of the cerebellar afferent circuit map. J. Neurosci. 30, 10015–10024 10.1523/JNEUROSCI.0653-10.2010 20668186 PMC2921890

[B158] Soriano P. (1999). Generalized lacZ expression with the ROSA26 Cre reporter strain. Nat. Genet. 21, 70–71 10.1038/5007 9916792

[B159] Sotelo C. (2004). Cellular and genetic regulation of the development of the cerebellar system. Prog. Neurobiol. 72, 295–339 10.1016/j.pneurobio.2004.03.004 15157725

[B160] Sotelo C. Bourrat F. Triller A. (1984). Postnatal development of the inferior olivary complex in the rat. II. Topographic organization of the immature olivocerebellar projection. J. Comp. Neurol. 222, 177–199 10.1002/cne.902220204 6321565

[B161] Sotelo C. Chedotal A. (1997). Development of the olivocerebellar projection. Perspect. Dev. Neurobiol. 5, 57–67 9509518

[B162] Sotelo C. Chedotal A. (2005). Development of the olivocerebellar system: migration and formation of cerebellar maps. Prog. Brain Res. 148, 1–20 10.1016/S0079-6123(04)48001-7 15661177

[B163] Sotelo C. Wassef M. (1991). Cerebellar development: afferent organization and Purkinje cell heterogeneity. Philos. Trans. R. Soc. Lond. B Biol. Sci. 331, 307–313 10.1098/rstb.1991.0022 1677476

[B164] Steinmayr M. Andre E. Conquet F. Rondi-Reig L. Delhaye-Bouchaud N. Auclair N. (1998). staggerer phenotype in retinoid-related orphan receptor alpha-deficient mice. Proc. Natl. Acad. Sci. U.S.A. 95, 3960–3965 9520475 10.1073/pnas.95.7.3960 PMC19945

[B165] Strata P. Scelfo B. Sacchetti B. (2011). Involvement of cerebellum in emotional behavior. Physiol. Res. 60(Suppl. 1), S39–S48 21777033 10.33549/physiolres.932169

[B166] Strick P. L. Dum R. P. Fiez J. A. (2009). Cerebellum and nonmotor function. Annu. Rev. Neurosci. 32, 413–434 10.1146/annurev.neuro.31.060407.125606 19555291

[B167] Sudarov A. Joyner A. L. (2007). Cerebellum morphogenesis: the foliation pattern is orchestrated by multi-cellular anchoring centers. Neural Dev. 2:26 10.1186/1749-8104-2-26 18053187 PMC2246128

[B168] Sudarov A. Turnbull R. K. Kim E. J. Lebel-Potter M. Guillemot F. Joyner A. L. (2011). Ascl1 genetics reveals insights into cerebellum local circuit assembly. J. Neurosci. 31, 11055–11069 10.1523/JNEUROSCI.0479-11.2011 21795554 PMC3153985

[B169] Sugihara I. (2005). Microzonal projection and climbing fiber remodeling in single olivocerebellar axons of newborn rats at postnatal days 4–7. J. Comp. Neurol. 487, 93–106 10.1002/cne.20531 15861456

[B170] Sugihara I. Bailly Y. Mariani J. (2000). Olivocerebellar climbing fibers in the granuloprival cerebellum: morphological study of individual axonal projections in the X-irradiated rat. J. Neurosci. 20, 3745–3760 10804216 10.1523/JNEUROSCI.20-10-03745.2000 PMC6772707

[B171] Sugihara I. Fujita H. Na J. Quy P. N. Li B. Y. Ikeda D. (2009). Projection of reconstructed single Purkinje cell axons in relation to the cortical and nuclear aldolase C compartments of the rat cerebellum. J. Comp. Neurol. 512, 282–304 10.1002/cne.21889 19003905

[B172] Sugihara I. Lang E. J. Llinas R. (1995). Serotonin modulation of inferior olivary oscillations and synchronicity: a multiple-electrode study in the rat cerebellum. Eur. J. Neurosci. 7, 521–534 7620604 10.1111/j.1460-9568.1995.tb00657.x

[B173] Sugihara I. Lohof A. M. Letellier M. Mariani J. Sherrard R. M. (2003). Post-lesion transcommissural growth of olivary climbing fibres creates functional synaptic microzones. Eur. J. Neurosci. 18, 3027–3036 10.1111/j.1460-9568.2003.03045.x 14656298

[B174] Sugihara I. Quy P. N. (2007). Identification of aldolase C compartments in the mouse cerebellar cortex by olivocerebellar labeling. J. Comp. Neurol. 500, 1076–1092 10.1002/cne.21219 17183552

[B175] Sugihara I. Shinoda Y. (2004). Molecular, topographic, and functional organization of the cerebellar cortex: a study with combined aldolase C and olivocerebellar labeling. J. Neurosci. 24, 8771–8785 10.1523/JNEUROSCI.1961-04.2004 15470143 PMC6729951

[B176] Sugihara I. Shinoda Y. (2007). Molecular, topographic, and functional organization of the cerebellar nuclei: analysis by three-dimensional mapping of the olivonuclear projection and aldolase C labeling. J. Neurosci. 27, 9696–9710 10.1523/JNEUROSCI.1579-07.2007 17804630 PMC6672958

[B177] Sugihara I. Wu H. Shinoda Y. (1999). Morphology of single olivocerebellar axons labeled with biotinylated dextran amine in the rat. J. Comp. Neurol. 414, 131–148 10.1002/(SICI)1096-9861(19991115)414:2<131::AID-CNE1>3.0.CO;2-F 10516588

[B178] Suzuki L. Coulon P. Sabel-Goedknegt E. H. Ruigrok T. J. (2012). Organization of cerebral projections to identified cerebellar zones in the posterior cerebellum of the rat. J. Neurosci. 32, 10854–10869 10.1523/JNEUROSCI.0857-12.2012 22875920 PMC6621006

[B179] Suzuki S. C. Inoue T. Kimura Y. Tanaka T. Takeichi M. (1997). Neuronal circuits are subdivided by differential expression of type-II classic cadherins in postnatal mouse brains. Mol. Cell. Neurosci. 9, 433–447 10.1006/mcne.1997.0626 9361280

[B180] Tanahira C. Higo S. Watanabe K. Tomioka R. Ebihara S. Kaneko T. (2009). Parvalbumin neurons in the forebrain as revealed by parvalbumin-Cre transgenic mice. Neurosci. Res. 63, 213–223 10.1016/j.neures.2008.12.007 19167436

[B181] Taniguchi H. He M. Wu P. Kim S. Paik R. Sugino K. (2011). A resource of Cre driver lines for genetic targeting of GABAergic neurons in cerebral cortex. Neuron 71, 995–1013 10.1016/j.neuron.2011.07.026 21943598 PMC3779648

[B182] Terada N. Banno Y. Ohno N. Fujii Y. Murate T. Sarna J. R. (2004). Compartmentation of the mouse cerebellar cortex by sphingosine kinase. J. Comp. Neurol. 469, 119–127 10.1002/cne.11002 14689477

[B183] Tsubota T. Ohashi Y. Tamura K. Sato A. Miyashita Y. (2011). Optogenetic manipulation of cerebellar Purkinje cell activity *in vivo*. PLoS ONE 6:e22400 10.1371/journal.pone.0022400 21850224 PMC3151259

[B184] Uemura T. Kakizawa S. Yamasaki M. Sakimura K. Watanabe M. Iino M. (2007). Regulation of long-term depression and climbing fiber territory by glutamate receptor delta2 at parallel fiber synapses through its C-terminal domain in cerebellar Purkinje cells. J. Neurosci. 27, 12096–12108 10.1523/JNEUROSCI.2680-07.2007 17978051 PMC6673370

[B185] Uesaka N. Mikuni T. Hashimoto K. Hirai H. Sakimura K. Kano M. (2012). Organotypic coculture preparation for the study of developmental synapse elimination in mammalian brain. J. Neurosci. 32, 11657–11670 10.1523/JNEUROSCI.1097-12.2012 22915109 PMC6703771

[B186] Ueyama T. Houtani T. Nakagawa H. Baba K. Ikeda M. Yamashita T. (1994). A subpopulation of olivocerebellar projection neurons express neuropeptide Y. Brain Res. 634, 353–357 10.1016/0006-8993(94)91943-7 8131087

[B187] Ugolini G. (1995). Specificity of rabies virus as a transneuronal tracer of motor networks: transfer from hypoglossal motoneurons to connected second-order and higher order central nervous system cell groups. J. Comp. Neurol. 356, 457–480 10.1002/cne.903560312 7642806

[B188] Van Der Giessen R. S. Maxeiner S. French P. J. Willecke K. De Zeeuw C. I. (2006). Spatiotemporal distribution of Connexin45 in the olivocerebellar system. J. Comp. Neurol. 495, 173–184 10.1002/cne.20873 16435305

[B189] Voogd J. Pardoe J. Ruigrok T. J. Apps R. (2003). The distribution of climbing and mossy fiber collateral branches from the copula pyramidis and the paramedian lobule: congruence of climbing fiber cortical zones and the pattern of zebrin banding within the rat cerebellum. J. Neurosci. 23, 4645–4656 12805304 10.1523/JNEUROSCI.23-11-04645.2003 PMC6740790

[B190] Voogd J. Ruigrok T. J. (2004). The organization of the corticonuclear and olivocerebellar climbing fiber projections to the rat cerebellar vermis: the congruence of projection zones and the zebrin pattern. J. Neurocytol. 33, 5–21 10.1023/B:NEUR.0000029645.72074.2b 15173629

[B190a] Wadiche J. I. Jahr C. E. (2005). Patterned expression of Purkinje cell glutamate transporters controls synaptic plasticity. Nat. Neurosci. 8, 1329–1334 10.1038/nn1539 16136036

[B191] Wall N. R. Wickersham I. R. Cetin A. De La Parra M. Callaway E. M. (2010). Monosynaptic circuit tracing *in vivo* through Cre-dependent targeting and complementation of modified rabies virus. Proc. Natl. Acad. Sci. U.S.A. 107, 21848–21853 10.1073/pnas.1011756107 21115815 PMC3003023

[B192] Wang V. Y. Rose M. F. Zoghbi H. Y. (2005). Math1 expression redefines the rhombic lip derivatives and reveals novel lineages within the brainstem and cerebellum. Neuron 48, 31–43 10.1016/j.neuron.2005.08.024 16202707

[B193] Wassef M. Chedotal A. Cholley B. Thomasset M. Heizmann C. W. Sotelo C. (1992). Development of the olivocerebellar projection in the rat: I. Transient biochemical compartmentation of the inferior olive. J. Comp. Neurol. 323, 519–536 10.1002/cne.903230405 1430320

[B194] Watanabe M. Kano M. (2011). Climbing fiber synapse elimination in cerebellar Purkinje cells. Eur. J. Neurosci. 34, 1697–1710 10.1111/j.1460-9568.2011.07894.x 22103426

[B195] Watanabe Y. Kinoshita K. Koguchi A. Yamamura M. (1997). A new method for evaluation of motor deficits in 3-acetylpyridine-treated rats. J. Neurosci. Methods 77, 25–29 10.1016/S0165-0270(97)00104-0 9402553

[B196] Watt A. J. Cuntz H. Mori M. Nusser Z. Sjostrom P. J. Hausser M. (2009). Traveling waves in developing cerebellar cortex mediated by asymmetrical Purkinje cell connectivity. Nat. Neurosci. 12, 463–473 10.1038/nn.2285 19287389 PMC2912499

[B197] Weible A. P. Schwarcz L. Wickersham I. R. Deblander L. Wu H. Callaway E. M. (2010). Transgenic targeting of recombinant rabies virus reveals monosynaptic connectivity of specific neurons. J. Neurosci. 30, 16509–16513 10.1523/JNEUROSCI.2442-10.2010 21147990 PMC3313909

[B198] Weickert S. Ray A. Zoidl G. Dermietzel R. (2005). Expression of neural connexins and pannexin1 in the hippocampus and inferior olive: a quantitative approach. Brain Res. Mol. Brain Res. 133, 102–109 10.1016/j.molbrainres.2004.09.026 15661370

[B199] White J. J. Reeber S. L. Hawkes R. Sillitoe R. V. (2012). Wholemount immunohistochemistry for revealing complex brain topography. J. Vis. Exp. 62:e4042 10.3791/4042 22508094 PMC3466652

[B200] White J. J. Sillitoe R. V. (2013). Development of the cerebellum: from gene expression patterns to circuit maps. WIREs Dev. Biol. 2, 149–164 10.1002/wdev.65 23799634

[B201] Wickersham I. R. Lyon D. C. Barnard R. J. Mori T. Finke S. Conzelmann K. K. (2007). Monosynaptic restriction of transsynaptic tracing from single, genetically targeted neurons. Neuron 53, 639–647 10.1016/j.neuron.2007.01.033 17329205 PMC2629495

[B202] Wickersham I. R. Sullivan H. A. Seung H. S. (2010). Production of glycoprotein-deleted rabies viruses for monosynaptic tracing and high-level gene expression in neurons. Nat. Protoc. 5, 595–606 10.1038/nprot.2009.248 20203674

[B203] Willson M. L. Bower A. J. Sherrard R. M. (2007). Developmental neural plasticity and its cognitive benefits: olivocerebellar reinnervation compensates for spatial function in the cerebellum. Eur. J. Neurosci. 25, 1475–1483 10.1111/j.1460-9568.2007.05410.x 17425573

[B204] Willson M. L. McElnea C. Mariani J. Lohof A. M. Sherrard R. M. (2008). BDNF increases homotypic olivocerebellar reinnervation and associated fine motor and cognitive skill. Brain 131, 1099–1112 10.1093/brain/awn024 18299295

[B205] Wilson S. L. Kalinovsky A. Orvis G. D. Joyner A. L. (2011). Spatially restricted and developmentally dynamic expression of engrailed genes in multiple cerebellar cell types. Cerebellum 10, 356–372 10.1007/s12311-011-0254-5 21431469 PMC3170510

[B206] Wise A. K. Cerminara N. L. Marple-Horvat D. E. Apps R. (2010). Mechanisms of synchronous activity in cerebellar Purkinje cells. J. Physiol. 588, 2373–2390 10.1113/jphysiol.2010.189704 20442262 PMC2915514

[B207] Woodward D. J. Hoffer B. J. Altman J. (1974). Physiological and pharmacological properties of Purkinje cells in rat cerebellum degranulated by postnatal x-irradiation. J. Neurobiol. 5, 283–304 10.1002/neu.480050402 4155719

[B208] Woodward D. J. Hoffer B. J. Lapham L. W. (1969). Postnatal development of electrical and enzyme histochemical activity in Purkinje cells. Exp. Neurol. 23, 120–139 10.1016/0014-4886(69)90039-9 5765001

[B209] Xiang M. Gan L. Zhou L. Klein W. H. Nathans J. (1996). Targeted deletion of the mouse POU domain gene Brn-3a causes selective loss of neurons in the brainstem and trigeminal ganglion, uncoordinated limb movement, and impaired suckling. Proc. Natl. Acad. Sci. U.S.A. 93, 11950–11955 8876243 10.1073/pnas.93.21.11950 PMC38164

[B210] Yamada M. Terao M. Terashima T. Fujiyama T. Kawaguchi Y. Nabeshima Y. (2007). Origin of climbing fiber neurons and their developmental dependence on Ptf1a. J. Neurosci. 27, 10924–10934 10.1523/JNEUROSCI.1423-07.2007 17928434 PMC6672857

[B211] Yamano M. Tohyama M. (1994). Distribution of corticotropin-releasing factor and calcitonin gene-related peptide in the developing mouse cerebellum. Neurosci. Res. 19, 387–396 8090368 10.1016/0168-0102(94)90080-9

[B212] Yan X. X. Garey L. J. (1996). Calretinin immunoreactivity in the monkey and cat cerebellum: cellular localisation and modular distribution. J. Hirnforsch. 37, 409–419 8872563

[B213] Zagrebelsky M. Strata P. Hawkes R. Rossi F. (1997). Reestablishment of the olivocerebellar projection map by compensatory transcommissural reinnervation following unilateral transection of the inferior cerebellar peduncle in the newborn rat. J. Comp. Neurol. 379, 283–299 10.1002/(SICI)1096-9861(19970310)379:2<283::AID-CNE9>3.0.CO;2-# 9050791

[B214] Zappala A. Parenti R. La Delia F. Cicirata V. Cicirata F. (2010). Expression of connexin57 in mouse development and in harmaline-tremor model. Neuroscience 171, 1–11 10.1016/j.neuroscience.2010.09.010 20849935

[B215] Zhang F. Wang L. P. Boyden E. S. Deisseroth K. (2006). Channelrhodopsin-2 and optical control of excitable cells. Nat. Methods 3, 785–792 10.1038/nmeth936 16990810

[B216] Zhu Y. Guthrie S. (2002). Expression of the ETS transcription factor ER81 in the developing chick and mouse hindbrain. Dev. Dyn. 225, 365–368 10.1002/dvdy.10166 12412022

